# Role of folding kinetics of secondary structures in telomeric G-overhangs in the regulation of telomere maintenance in *Saccharomyces cerevisiae*

**DOI:** 10.1074/jbc.RA120.012914

**Published:** 2020-05-08

**Authors:** Katarina Jurikova, Martin Gajarsky, Mona Hajikazemi, Jozef Nosek, Katarina Prochazkova, Katrin Paeschke, Lukas Trantirek, Lubomir Tomaska

**Affiliations:** 1 Department of Genetics, Faculty of Natural Sciences, Comenius University in Bratislava, Bratislava, Slovakia; 2 Central European Institute of Technology, Masaryk University, Brno, Czech Republic; 3 Department of Oncology, Hematology and Rheumatology, University Hospital Bonn, Bonn, Germany; 4 Department of Biochemistry, Faculty of Natural Sciences, Comenius University in Bratislava, Bratislava, Slovakia; 5 Institute of Biophysics, Czech Academy of Sciences, Brno, Czech Republic

**Keywords:** telomere, telomerase, Saccharomyces cerevisiae, cell cycle, Cdc13, G-hairpin, G-quadruplex, folding kinetics

## Abstract

The ends of eukaryotic chromosomes typically contain a 3′ ssDNA G-rich protrusion (G-overhang). This overhang must be protected against detrimental activities of nucleases and of the DNA damage response machinery and participates in the regulation of telomerase, a ribonucleoprotein complex that maintains telomere integrity. These functions are mediated by DNA-binding proteins, such as Cdc13 in *Saccharomyces cerevisiae*, and the propensity of G-rich sequences to form various non-B DNA structures. Using CD and NMR spectroscopies, we show here that G-overhangs of *S. cerevisiae* form distinct Hoogsteen pairing–based secondary structures, depending on their length. Whereas short telomeric oligonucleotides form a G-hairpin, their longer counterparts form parallel and/or antiparallel G-quadruplexes (G4s). Regardless of their topologies, non-B DNA structures exhibited impaired binding to Cdc13 *in vitro* as demonstrated by electrophoretic mobility shift assays. Importantly, whereas G4 structures formed relatively quickly, G-hairpins folded extremely slowly, indicating that short G-overhangs, which are typical for most of the cell cycle, are present predominantly as single-stranded oligonucleotides and are suitable substrates for Cdc13. Using ChIP, we show that the occurrence of G4 structures peaks at the late S phase, thus correlating with the accumulation of long G-overhangs. We present a model of how time- and length-dependent formation of non-B DNA structures at chromosomal termini participates in telomere maintenance.

## Introduction

More than 80 years ago, Herman Muller and Barbara McClintock predicted that terminal regions of linear chromosomes harbor special functions, and they termed these regions telomeres (1, 2). Their visionary studies showed that telomeres are vital protective caps that prevent nucleolytic degradation of chromosomal ends and their erroneous recognition as double-strand breaks by DNA repair machinery (3). In addition to this end-protection problem, telomeres must address the end-replication problem caused by the inability of conventional DNA polymerases to complete DNA replication at chromosomal termini. The incomplete synthesis of terminal DNA regions of the chromosomes, referred to as telomeric DNA, results in the shortening of DNA followed by cell senescence and chromosomal instability (4–6).

Telomeric DNA of eukaryotic nuclear chromosomes provides a platform for the assembly of molecular partners involved in mediating telomere functions. In the case of eukaryotic nuclear chromosomes, this platform usually consists of a dsDNA region composed of short GC-rich tandem repeats, terminating with a G-rich 3′ ssDNA overhang (G-overhang). Many organisms possess a subtle variation of a vertebrate telomeric repeat of 5′-TTAGGG-3′ (7); however, these repeats may be of variable lengths (8) or heterogeneous in sequence, and this is the case in the yeast *Saccharomyces cerevisiae* (9). The length of the G-overhang exhibits interspecific, intercellular, and interchromosomal variations (10, 11). Telomeric DNA is bound by a set of dedicated proteins forming a protective nucleoprotein structure comprising a number of telomere-binding proteins that often exhibit binding specificity (3, 12). Examples include the dsDNA-binding protein Rap1 and the G-overhang–binding protein Cdc13 in *S. cerevisiae*. Telomere shortening is most often prevented via extension by telomerase, a ribonucleoprotein enzymatic complex that includes an RNA template and a reverse transcriptase subunit, followed by the fill-in synthesis of the C-rich strand mediated by primase, Pol α, which maintains the number of tandem repeats within a relatively stable interval (13–16).

The other means of protecting the very tip of the chromosome is based on inherent evolutionarily conserved characteristics of the G-overhang (*i.e.* its ability to fold into non-B DNA structures, which are considered as its universal epigenetic hallmark) (17). The G-rich telomeric DNA from the vast majority of eukaryotes displays a propensity to form a four-stranded structure called a G-quadruplex (G4) (10, 18–20) that relies on the formation of planar guanine tetrads (G-quartets) marked by a Hoogsteen base-pairing-type guanine-guanine pattern. G4 formation at telomeres has been demonstrated to alter telomerase activity (21–25). Currently, controversial data exist that link G4 formation and function to telomeres. In detail, antiparallel G4 structures block telomerase (24), whereas intramolecular parallel G4 structures support telomerase binding to telomeres (21, 22). Telomeric G4 structures interact with a number of telomere-associated proteins. Helicases involved in telomere maintenance, Sgs1 in *S. cerevisiae*, human WRN and BLM, and widely conserved Pif1, all bind and unwind G4 structures (26–31). Est1, a subunit of budding yeast telomerase involved in the recruitment of the complex to a G-overhang, was shown to bind G4 structures and promote their formation (32, 33). Human telomerase, which also displays G4 binding, was found to partially unwind and extend the G4 structure (34). Additionally, observations that the processivity of telomerase and association with telomeres can be impaired by ligands stabilizing telomeric G4 structures both *in vitro* and *in vivo* not only marked telomeric G4 structures as potential molecular targets suitable for anticancer therapy but also further demonstrated that G4 formation is an essential component for telomere function and maintenance (34–38). Additionally, G4 also interacts with telomere-binding proteins; in *S. cerevisiae*, Rap1 stimulates G4 formation *in vitro* (39, 40), and a functional interplay between the formation of G4 structures and Cdc13 binding has been suggested by both *in vitro* and *in vivo* experiments (41, 42). These data suggest a link between G4 and telomere function in budding yeast; however, the mechanistic details of their participation remain elusive.

In vertebrates, including humans, the length of telomeric G-overhangs fluctuates between 50 and 250 nt during the cell cycle (43, 44). As only four human telomeric DNA tandem repeats are required for the formation of a G4 structure (45), the capacity to form a G4 is maintained throughout the whole cell cycle. In contrast, in *S. cerevisiae*, the G-overhang is rather short (9–14 nt) throughout most of the cell cycle and is extended to 30–100 nt only in the late S phase (46, 47). As the capacity to form a G4 structure depends on the length of the ssDNA (17, 19), the overhangs are presumably unable to form a G4 during most of the cell cycle. However, shorter telomeric ssDNA may adopt an alternative structure. Recently, our group demonstrated that the abundant short (11-nt) sequence motif (5′-GTGTGGGTGTG-3′), covering more than 90% of irregular telomeric DNA in *S. cerevisiae*, folds into a novel DNA structure that is a mixture of parallel/antiparallel fold-back structures stabilized by guanine-guanine base pairs, herein referred to as a G-hairpin (48).

In this study, we investigated the stereochemical properties of model oligonucleotides emulating telomeric G-overhangs of different lengths with regard to the kinetics of non-B DNA structure formation, their folding topologies, and their capacity to interact with Cdc13. We found that the formation of a stable secondary structure in both long and short telomeric G-overhangs impairs the binding to Cdc13. Additionally, whereas the formation of non-B DNA structures in a long telomeric G-overhang proceeds on the time scale of relevant biological processes (*e.g.* the S phase), the time required for the formation of a G-hairpin structure notably exceeds the duration of the cell cycle. Our results suggest that the physiological roles of non-B DNA structures in telomeric G-overhangs are to tune the interaction between Cdc13 and telomeric DNA. We propose a kinetically based model for the initial phase of the telomerase catalytic cycle involving the recruitment of Cdc13 to a telomeric G-overhang. In this model, the folding kinetics of non-B DNA structures of G-overhangs play the role of a switch in the control of Cdc13 binding to a G-overhang, indicating that it may be involved in telomerase recruitment.

## Results

### Oligonucleotides of different lengths emulating telomeric ssDNA overhangs from S. cerevisiae fold into intramolecular G4 structures with different topologies

The oligonucleotide-emulating short telomeric ssDNA overhang (11 nt) of *S. cerevisiae* (ONG11) was recently shown to fold into an intramolecular G-hairpin (48). In contrast, previous CD and NMR studies on oligonucleotides emulating extended (>19-nt) telomeric DNA indicated the formation of G4 structures (17, 19). As the folding topology of a G4 is known to strongly depend on the nucleotide composition and length of the investigated fragment, we conducted an analysis of 10 different DNA constructs corresponding to various lengths with 21–33-nt-long truncations derived from the native (irregular) telomeric DNA of *S. cerevisiae* (Table 1) using CD spectroscopy and nondenaturing PAGE.

**Table 1:** **List of oligonucleotide constructs employed for screening the conformational space of extended telomeric ssDNA from *S. cerevisiae*. Structural types and topologies of non-B DNA structures were assigned on the basis of CD spectra (Fig. 1)**

	Length	Sequence (5′ → 3′)	Structural type	Dominant topology
	*nt*			
ONG1	33	GTGTGGGTGTGGTGTGGGTGTGGTGTGGGTGTG	G-quadruplex	Parallel
ONG2	22	GTGTGGGTGTGGTGTGGGTGTG	G-quadruplex	Parallel
ONG3	32	GTGTGGGTGTGGTGTGGGTGTGGTGTGGGTGT	G-quadruplex	Parallel
ONG4	32	TGTGGGTGTGGTGTGGGTGTGGTGTGGGTGTG	G-quadruplex	Parallel
ONG5	30	GTGTGGGTGTGGTGTGGGTGTGGTGTGGGT	G-quadruplex	Parallel
ONG6	30	TGGGTGTGGTGTGGGTGTGGTGTGGGTGTG	G-quadruplex	Parallel
ONG7	27	TGGGTGTGGTGTGGGTGTGGTGTGGGT	G-quadruplex	Mixture
ONG8	26	TGTGGTGTGGGTGTGGTGTGGGTGTG	G-quadruplex	Mixture
ONG9	21	TGGGTGTGGTGTGGGTGTGGT	G-quadruplex	Antiparallel
ONG10	21	TGGTGTGGGTGTGGTGTGGGT	G-quadruplex	Antiparallel
ONG11	11	GTGTGGGTGTG	G-hairpin	

**Figure 1.**
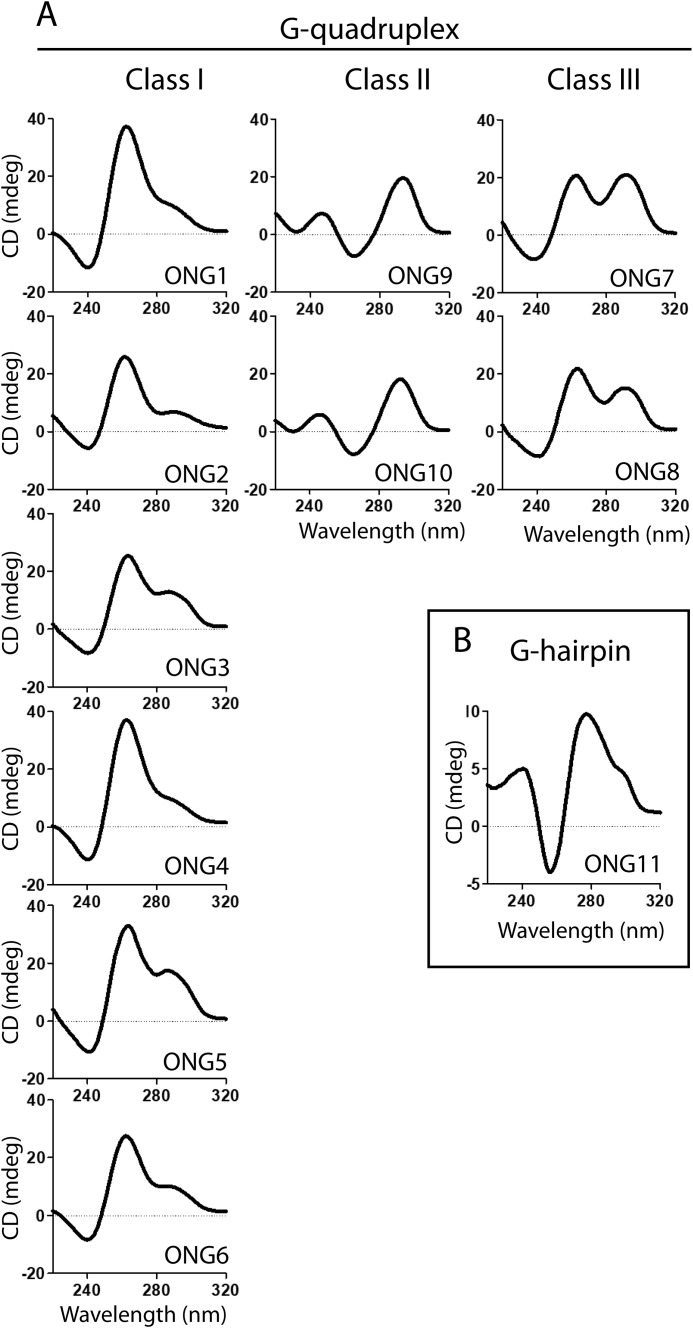
**Oligonucleotides emulating long and short telomeric G-overhangs adopt distinct non-B DNA structures.** CD spectra of oligonucleotide constructs emulating long (*A*) and short (*B*) telomeric G-overhangs (*cf*. Table 1). CD spectra were measured at RT in K^+^S buffer at a DNA concentration of 50 μm. The spectra were measured 24 h after sample annealing.

As seen in Fig. S1, the migration rates of all the constructs in nondenaturing PAGE were consistent with the formation of monomolecular species. The shapes of the CD spectra, acquired 24 h after annealing, were indicative of the formation of G4 structures for all the constructs. Based on the shapes of the CD spectra, the individual constructs were clustered into three distinct classes (Fig. 1). Class I (ONG1–6) displayed CD spectra marked by two dominant bands, one negative at ∼240 nm and one positive at ∼260 nm; this is the characteristic spectrum of a parallel G4. Class II (ONG9 and ONG10) displayed CD spectra with a dominant negative band at ∼265 nm and a positive band at ∼290 nm; this is a characteristic spectrum of an antiparallel G4. Class III (ONG7 and ONG8) displayed CD spectra with two dominant positive bands at ∼260 and ∼290 nm, indicating that the constructs exist as a mixture of parallel and antiparallel G4 structures. Note, however, that although the shapes of the CD spectra of ONG1-6 and ONG9-10 were indicative of having parallel and antiparallel G4 structures, respectively, the detailed analysis of spectral data revealed that these constructs are similar to ONG7 and ONG8 and capable of adopting at least two distinct G4 topologies that co-exist as a mixture of dominant and minor G4 species (for the detailed analysis of ONG1 and ONG9, which are representative of Class I and Class II constructs, see the next paragraph). Altogether, the presented data indicate that whereas short telomeric overhangs that are present at telomeres for the majority of the cell cycle have the propensity to form G-hairpin (48), the extended telomeric overhangs in the late S phase have the capacity to fold into topologically diverse G4 structures.

### Distinct secondary structures in short and extended telomeric overhangs have similar thermodynamic stabilities, but they notably differ in their folding kinetics

**Figure 2.**
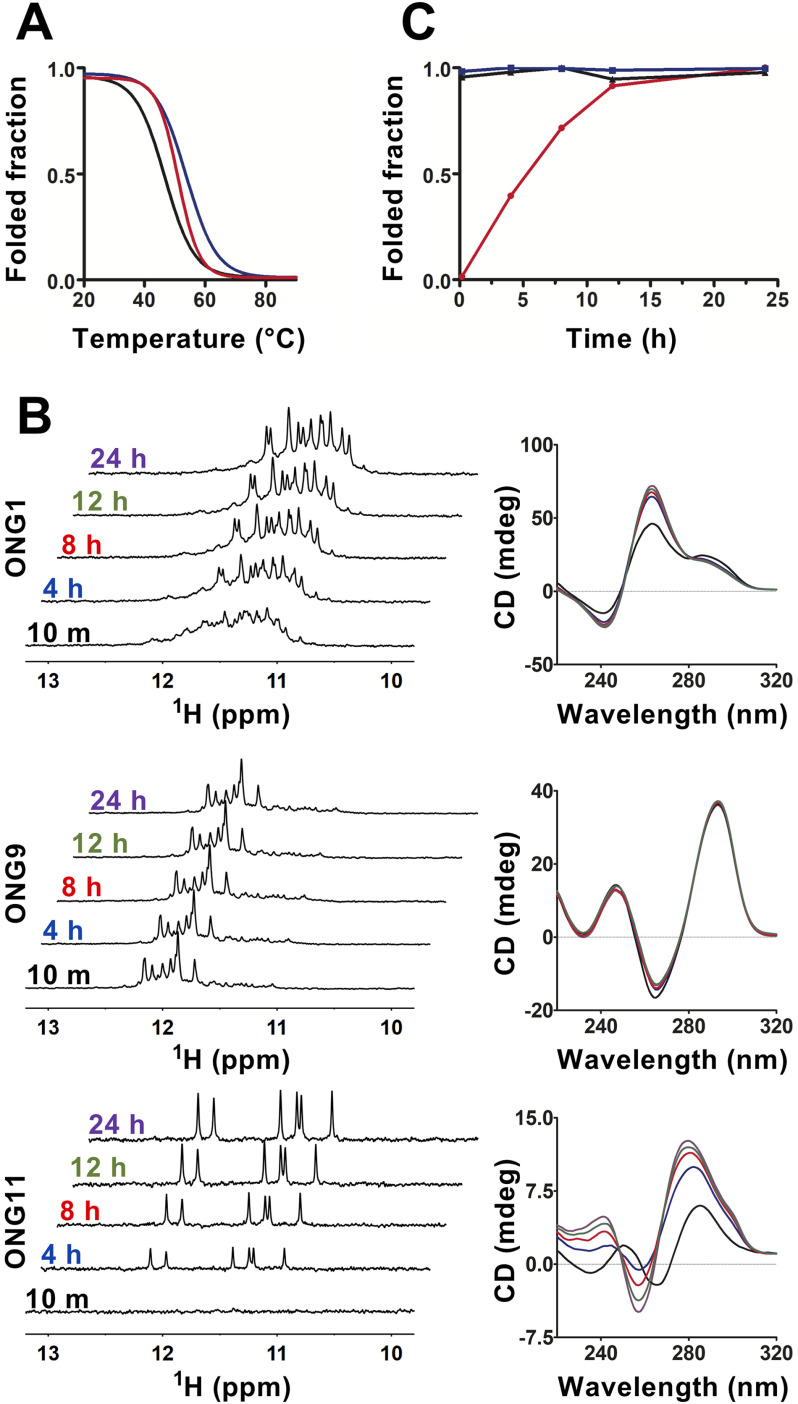
**Non-B DNA structures in long and short telomeric G-overhangs have notably distinct formation kinetics.** *A*, normalized CD melting curves for ONG1 (*black*; apparent *T_m_* = 46.3 °C), ONG9 (*blue*; apparent *T_m_* = 53.5 °C), and ONG11 (*red*; apparent *T_m_* = 50.7 °C). *B*, imino region of 1D ^1^H NMR (*left*) and CD (*right*) spectra of ONG1, ONG9, and ONG11 acquired as a function of time (time points indicated). The NMR and CD spectra were measured in K^+^S buffer at a DNA concentration of 50 μm. NMR spectra were acquired using a zggpw5 pulse sequence (see “Experimental procedures”). *C*, time course of the folding process for ONG1 (*black*, *triangle*), ONG9 (*blue*, *square*), and ONG11 (*red*, *circle*) as estimated from the normalized time-dependent changes of imino signals in NMR spectra presented in *A*.

As any biological action of DNA secondary structures is jointly governed not only by conformational features but also by thermodynamic and kinetics factors, we set out to characterize the thermodynamic stability and estimate the folding kinetics for (i) the DNA G-hairpin being representative of a secondary structural element in a short telomeric overhang (ONG11) and (ii) parallel and antiparallel G4 structures forming in the model of an extended telomeric overhang (ONG1). However, as the antiparallel form cannot be kinetically isolated within the ONG1 context, we used ONG9, which almost exclusively adopts an antiparallel G-quadruplex structure (see below), as a model emulating antiparallel G-quadruplex that is forming in the context of a long telomeric overhang. To obtain thermodynamic stability information, the CD melting profiles for ONG1, ONG9, and ONG11 were acquired at pH 7.2 in K^+^S buffer. As shown in Fig. 2*A*, the melting temperatures for these structures were markedly similar, ranging from 46.3 to 53.5 °C. The acquired melting profiles indicate that formation of G-hairpin in a short telomeric overhang as well as antiparallel and parallel G-quadruplex structures in an extended telomeric overhang is plausible under physiologically relevant temperatures.

Despite the different appearances in the CD spectra of ONG1 and ONG9 (Fig. 1), the time-resolved CD and NMR measurements revealed similarities in their folding. The NMR spectrum of ONG1 acquired at *t* = 10 min was unresolved and characteristic of a polymorphic mixture of at least two G4 species, whereas the NMR spectrum of ONG1 acquired at *t* = 4 h displayed ∼12 resolved signals (the number expected for a single three-G-tetrad-based G4 structure) (see Fig. 2*B*). These time-dependent changes in the NMR spectral pattern are a characteristic indication of the kinetic control of G4 formation, where the kinetically preferred G4 topology is distinct from that corresponding to the thermodynamically preferred state (49). Hyperchromic and hypochromic shifts in the CD spectra of ONG1 observed at ∼260 and ∼285 nm, respectively, between *t* = 10 min and *t* = 4 h (Fig. 2), suggested both the antiparallel and parallel G4 structures as being the kinetically and thermodynamically preferred conformations, respectively. The behavior of ONG9 seemingly differed from that of ONG1; the NMR spectra displayed two independent sets of imino signals regardless of the data acquisition time: eight strong well-resolved signals in the region of 11.6-12.3 ppm (from dominant species) and ∼12 very weak signals (from minor species) in the region of 10.8-11.6 ppm (Fig. 2*B*). Whereas the number of signals in the region of 11.6-12.3 ppm was consistent with that expected for a G4 consisting of two G-tetrads, the number of signals from the minor species was consistent with a G4 structure consisting of three G-tetrads. The overall appearance of the NMR and CD spectra (Fig. 2) indicated that the ONG9 folds into a mixture of dominant two-tetrad antiparallel and (minor) three-tetrad–based G4 structure. Based on the NMR spectra, the population of the minor species was estimated to be ∼5% (Fig. 2*B*). Importantly, the spectral signatures of both NMR and CD spectra for ONG9 were found to be time-independent (Fig. 2*B*). This suggests that both dominant and minor species have virtually identical thermodynamic stabilities and that the formation of two-tetrad and three-tetrad G4 structures is, similar to the situation with ONG1, under kinetic control (with a folding rate for the two-tetrad G4 being faster than that for the three-tetrad G4).

Most importantly, the folding of G4 structures (ONG1 and ONG9) proceeded on a time scale dramatically faster than that of the G-hairpin (ONG11). In contrast to the NMR spectrum for ONG9, which was fully developed at *t* = 10 min, the corresponding spectrum of ONG11 was devoid of any signals, indicating the absence of a folded species (G-hairpin) (Fig. 2*B*). Although imino signals characteristic of G-hairpin formation were detected at *t* = 4 h, their intensities further increased over time, suggesting an ongoing folding process (Fig. 2*B*). Notably, the time-dependent characteristics of the changes in the NMR spectra of ONG11 were fundamentally distinct from those observed for ONG1. Although the pattern of the NMR spectrum of ONG1 also changed with time, the integral intensities of the imino signals remained constant (Fig. 2*C*), indicating that the changes in the NMR spectral pattern were not due to changes in the populations of unfolded and folded species but rather due to the refolding of a kinetically preferred G4 structure into a thermodynamically preferred G4 structure (*i.e.* a process that presumes a steady population of the unfolded state) (49). Altogether, the time-resolved CD and NMR data corroborated that G4 structures folded on a time scale that is comparable with or faster than 10 min (resolution limit of our experiment), whereas the G-hairpin folding proceeded on a time scale of several hours (Fig. 2*C*).

To assess the relevance of these *in vitro* data to *in vivo* physiological situations, where the folding of these structures might be actively modulated by cellular factors, we compared the folding of ONG1 (a model of a G-quadruplex–forming telomeric overhang), and ONG11 (a model of a G-hairpin–forming short telomeric overhang) in crude yeast cell lysate. Individual constructs were annealed in buffer and then mixed with the cell lysate immediately upon cooling down to room temperature. Whereas the 1D ^1^H NMR spectrum of ONG1 acquired in the presence of the cell lysate showed, similarly to the situation in the buffer, a characteristic pattern for G4 formation at 10 min, the respective spectrum of ONG11 (G-hairpin) was devoid of any imino signals (Fig. S2). The absence of the imino signals in the lysate NMR spectra of ONG11 suggests either that ONG1 remains unfolded at the timescale of the NMR experiment or that ONG11 is bound by high-molecular weight cellular factors (causing relaxation broadening of NMR signals). In either case, these data argue against the existence of the intracellular factors promoting G-hairpin folding, although such activities may be present in intact cells. Together, these results suggest that the formation of secondary structures in short and extended telomeric overhangs is kinetically rather than thermodynamically resolved; in addition, both the structural and formation kinetics should be considered when attempting to assess their relevance *in vivo*.

### Secondary structures in telomeric DNA, regardless of their topology, interfere with Cdc13 binding

Cdc13 binds to G-overhang to mediate the recruitment of telomerase to telomeric DNA (50–52). Analogously to the length of the G-overhang, the activity of telomerase is regulated by the cell cycle. Telomerase is recruited to telomeres in the late S phase, a stage also marked by the presence of long (>30-nt) G-overhangs, resulting from both telomerase action and nuclease resection of the 5′ strand (46, 53). The relationship between the telomerase recruitment and length-dependent folding of a G-overhang into DNA secondary structures is currently unknown. As our previous experiments have shown that different secondary structures form on G-overhangs of different lengths with different folding kinetics, the ability of Cdc13 to bind to the secondary structures formed by G-overhangs may influence the presence of Cdc13 on the overhang and thus also may impact telomerase recruitment to telomeres.

**Figure 3.**
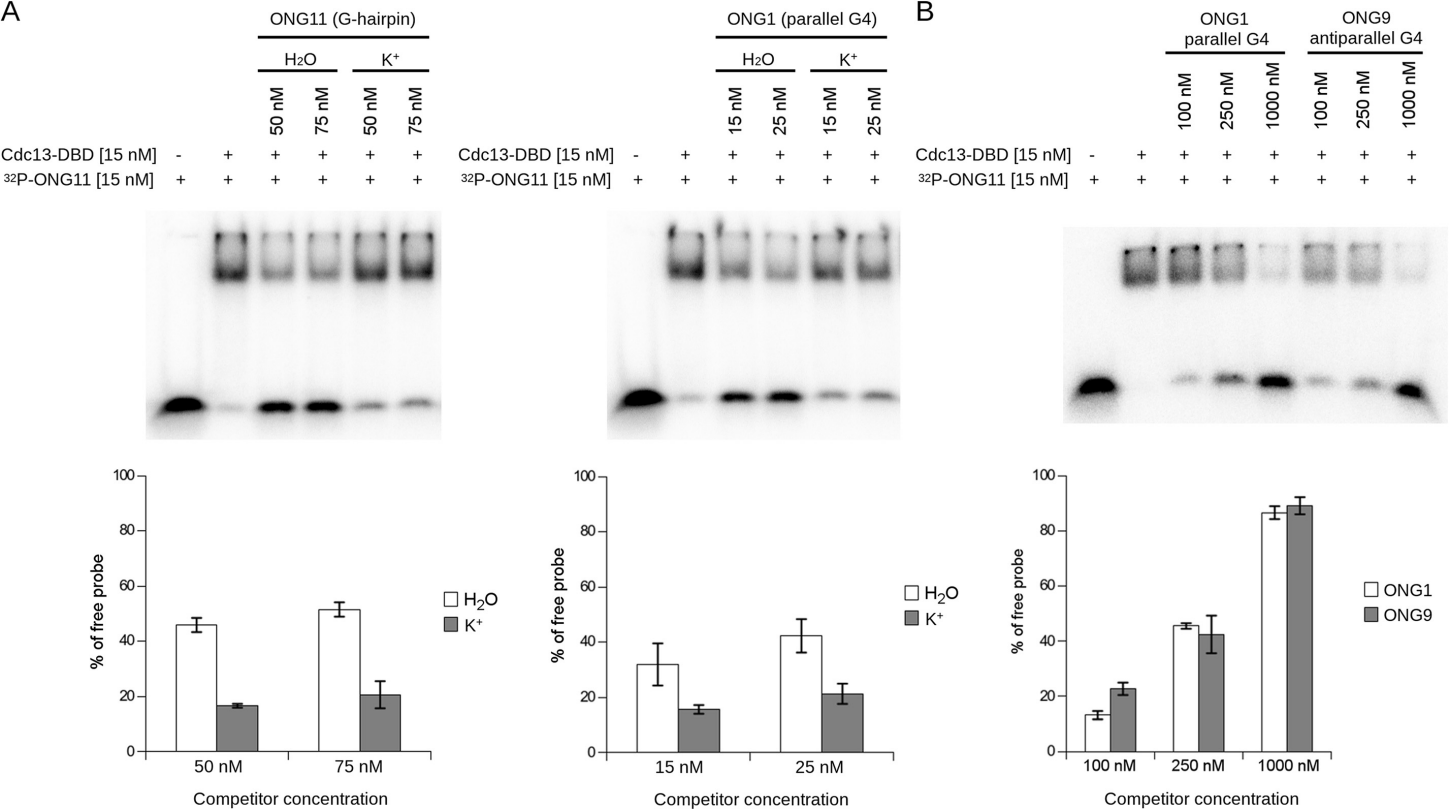
**Secondary structures forming on telomeric DNA decrease the binding of Cdc13-DBD.** *A*, *top*, EMSA with radioactively labeled ONG11 as a probe and ONG11 and ONG1 as unlabeled competitors. For the reactions with folded competitors (*labeled K*^+^), the unlabeled DNA oligonucleotides were diluted in 1× K^+^ buffer, boiled, allowed to cool, incubated at 22 °C for 72 h, mixed with other components of the EMSA reaction to a final concentration of 50 or 75 nm, incubated for 10 min at 22 °C, and loaded onto the gel. For the reactions with unfolded competitors (*labeled H_2_O*), the unlabeled DNA oligonucleotides were diluted in water, boiled, allowed to cool, immediately mixed with other components of the EMSA reaction to a final concentration of 50 or 75 nm, incubated for 10 min at 22 °C, and loaded onto the gel. The labeled probe was boiled and cooled immediately prior to adding to EMSA reactions. For both oligonucleotides, the folded competitors exhibit decreased competing ability (measured as the amount of freely migrating probe) compared with the controls in water. *Bottom*, quantification of the free probe, mean ± S.D.; *n* = 4 and *n* = 3, independent replicas for ONG1 and ONG11, respectively. *B*, the G4 topology does not impact Cdc13-DBD binding. *Top*, EMSA with radioactively labeled ONG11 as a probe and folded ONG1 (parallel G4) and ONG9 (antiparallel G4) as competitors. The unlabeled DNA oligonucleotides were diluted in 1× K^+^ buffer, boiled, incubated at 22 °C for 24 h, and then mixed with other components of the EMSA reaction to a final concentration of 100, 250, or 1000 nm. The labeled probe was boiled and cooled immediately prior to adding to the EMSA reactions. *Bottom*, quantification of the free probe, mean ± S.D., *n* = 2, independent replicas. *Error bars*, S.D.

To address this issue, we assessed the ability of Cdc13 to bind telomeric oligonucleotides exhibiting various secondary structures *in vitro*. For the experiments, we employed the principal oligonucleotide/oligosaccharide-binding (OB) fold domain of Cdc13 spanning residues 497–694 (Cdc13-DBD), whose DNA-binding properties in terms of dissociation constants are very similar to those of the full-length Cdc13 (*K_D_* of Cdc13-DBD for ONG11 is 0.25-0.5 of the *K_D_* of Cdc13 (54)). We expressed Cdc13-DBD in the *Escherichia coli* BL21(DE3) strain and purified the recombinant protein using affinity chromatography (Fig. S3). The binding properties of the purified protein were assessed and found to correspond to previously published studies (Fig. S4) (55, 56). The binding between Cdc13-DBD and ONG1, ONG9, or ONG11, which are representative of telomeric parallel G4, antiparallel G4, and G-hairpin structures, respectively (Table 1), was assessed with respect to the ssDNA (unfolded) G-hairpin–forming sequence of 5′-GTGTGGGTGTG-3′ (ONG11), which binds to the Cdc13-DBD with high affinity (54). For the purpose of the assay, the preformed complex between Cdc13-DBD and an unlabeled competitor (ONG1, ONG9, or ONG11) was incubated with a radioactively ^32^P-labeled probe (ONG11), and the capacity of the unlabeled oligonucleotides to outcompete the binding of the ^32^P-labeled ONG11 was assessed by observing the amount of freely migrating labeled probe on a gel. In each experiment, the competitor DNA was annealed and incubated for the corresponding time in either water (favoring the unfolded state) or 1× K^+^ buffer, which favors folding into secondary structures (Fig. S5). The results of electrophoretic mobility shift assays (EMSAs) showed that the competitor oligonucleotides in the folded form (K^+^) exhibit a decreased ability to displace the labeled probe from binding to Cdc13-DBD when compared with their unfolded counterparts (Fig. 3*A*). To further confirm that the displaced free probe is due to the presence of the competitor DNA and not due to other reaction components, we also tested a wider range of competitor concentrations (Fig. S6). These results indicate that for both G-hairpin (ONG11) and G4 (ONG1), Cdc13-DBD has lower affinity toward the secondary structures formed on the telomeric DNA than toward the same oligonucleotides in their ssDNA form.

**Figure 4.**
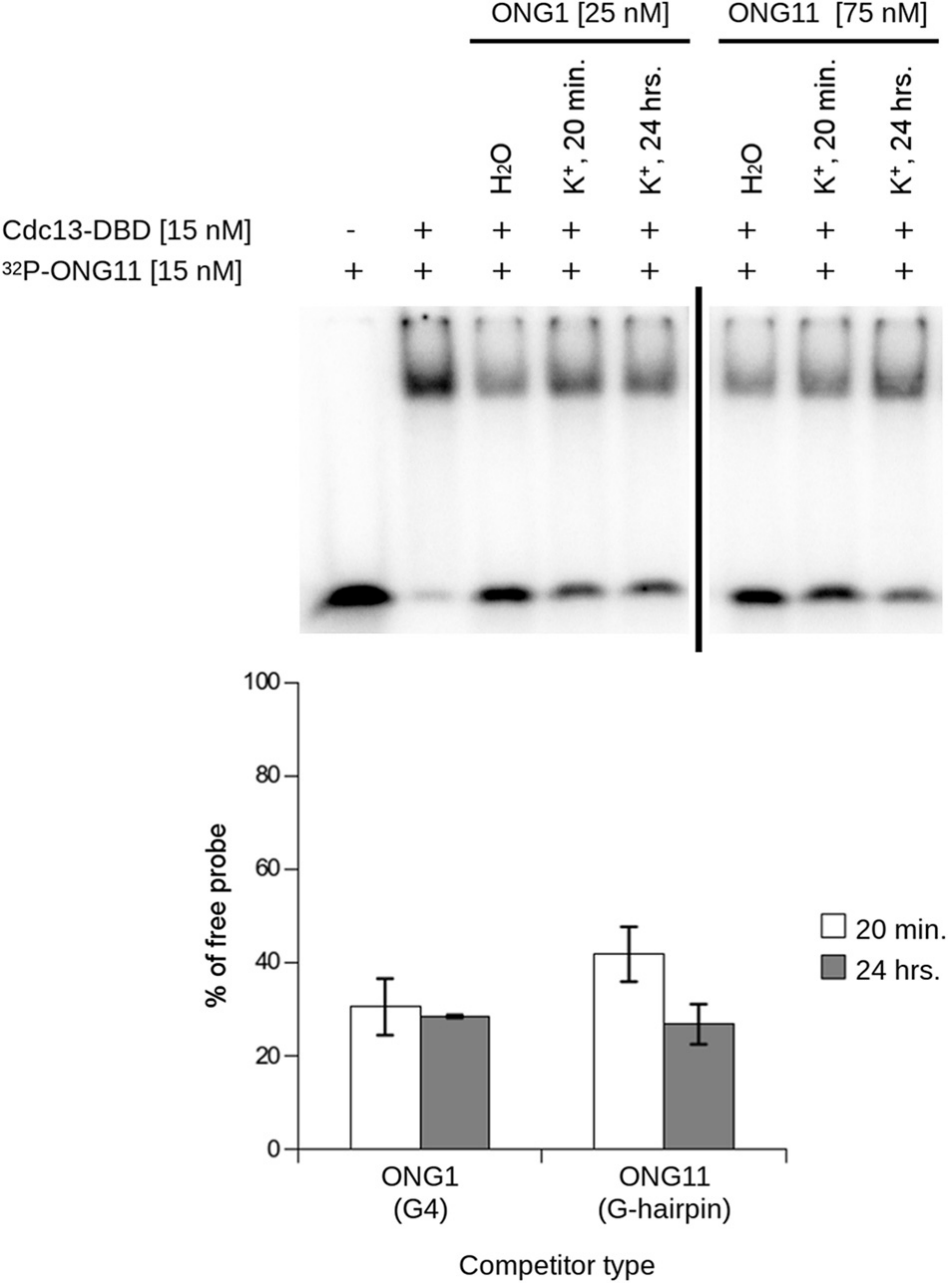
**Different folding kinetics of G-hairpin and G-quadruplex affect their ability to bind Cdc13-DBD in a time-dependent manner.** *Top*, EMSA with radioactively labeled ONG11. ONG1 and ONG11 were used as unlabeled competitors that were allowed to fold in 1× K^+^ buffer for the indicated time periods. Whereas ONG11 (G-hairpin) shows decreased binding of Cdc13-DBD between 20 min and 24 h, ONG1 (parallel G4) does not exhibit this time-dependent change. *Bottom*, quantification of the free probe, mean ± S.D., *n* = 2, independent replicas. ONG1 and ONG11 were analyzed on separate gels, as indicated by the *vertical bar*. *Error bars*, S.D.

**Figure 5.**
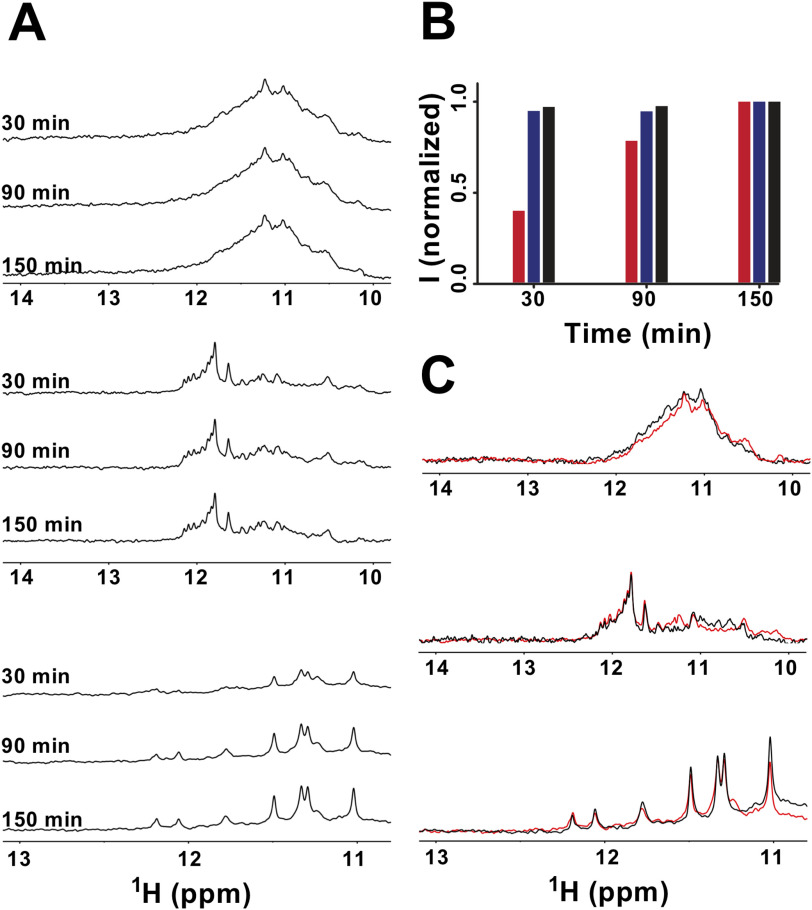
**Cdc13-DBD does not form a stable complex with either a G-hairpin or a G-quadruplex and does not interfere with their folding kinetics.** *A*, imino regions of the 1D ^1^H NMR spectra of an equimolar mixture of ONG1 (*top*), ONG9 (*middle*), ONG11 (*bottom*), and Cdc13-DBD were acquired as a function of time (indicated). *B*, time course of the folding process for ONG1 (*black box*), ONG9 (*blue box*), and ONG11 (*red box*) in the presence of equimolar amounts of Cdc13-DBD as estimated from normalized time-dependent changes in the intensity of imino signals (*I*) from NMR spectra presented in *A*. The imino signal intensities at *t* = 30 and *t* = 90 min were normalized with respect to those acquired at *t* = 150 min. The NMR spectra were acquired using a 1-1 echo pulse sequence with an excitation maximum set to 12 ppm. *C*, overlay of the imino regions of the 1D ^1^H NMR spectra of ONG1 (*top*), ONG9 (*middle*), and ONG11 (*bottom*) measured in K^+^TD buffer in the absence (*black*) and in the presence of equimolar amounts of Cdc13-DBD (*red*).

**Figure 6.**
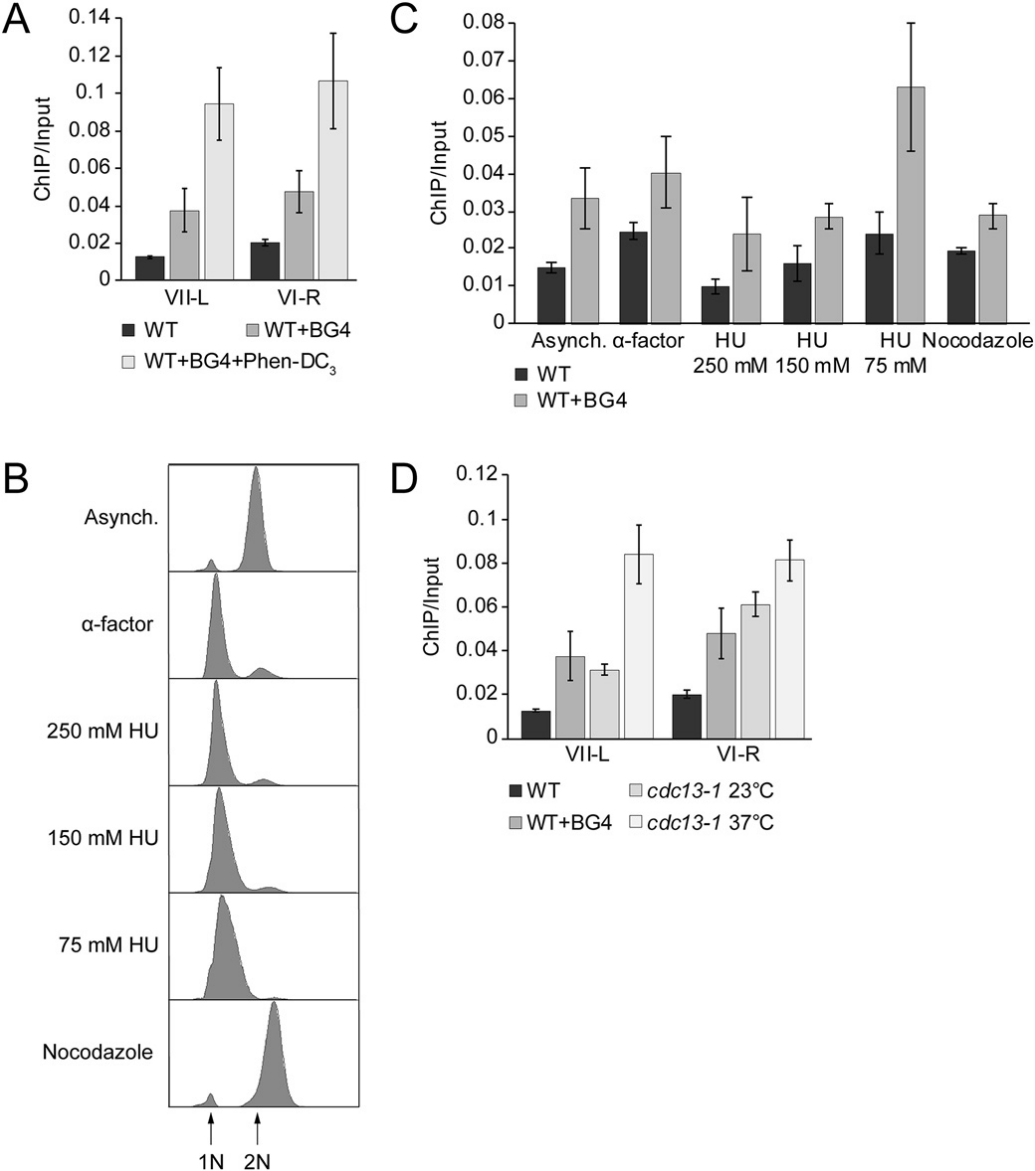
**Formation of telomeric G-quadruplexes changes during the cell cycle in *S. cerevisiae*.** *A–D*, formation of G4 at telomeres was monitored by BG4 ChIP and analyzed by qPCR. G4 levels were detected in WT yeast cells. *A*, BG4 ChIP and qPCR analysis at two different telomeres (telomere VII-L and VI-R). As control, ChIP and qPCR were performed in the absence of BG4 antibody and 10 μm Phen-DC_3_ to determine the specific binding of BG4. Data were normalized to input material (*ChIP/Input*). *B*, FACS analysis of yeast strains to monitor the arrest in specific cell cycle phases. Cells were arrested in G_1_ phase with α factor for 3 h. For different S-phase time points, cells were released from G_1_ in the presence of the indicated concentrations of HU (early S, 250 mm HU; mid-S phase, 150 mm HU; and late S phase, 75 mm HU). For G_2_ phase, cells were treated with 15 µg/ml nocodazole for 2 h. *C*, BG4 ChIP and qPCR analysis to monitor G4 levels in different cell cycle phases at telomere VII-L. BG4 ChIP signals were normalized to input. Plotted are the means of at least three biological replicates. *Error bars* represent SEM. Significance was calculated based on Student's *t* test comparing 75 mm HU with the other cell cycle phases. G4 level at 75 mm HU compared with other cell cycle phases was significantly higher according to the *t* test (*p* < 0.02). Only in G_1_ arrest (α factor), a significance of *p* = 0.1 was calculated. *D*, BG4 ChIP and qPCR analysis at two different telomeres (telomere VII-L and VI-R) in *cdc13-1* mutant at nonpermissive temperature (37 °C). As control, the BG4 ChIP was done in *cdc13-1* mutant at permissive temperature (23 °C) and WT strain. We observed 5-6-fold higher enrichment of telomeric G4s in *cdc13-1* strain at nonpermissive temperature compared with WT. Data were normalized to input material (*ChIP/Input*).

Previously, parallel and antiparallel G4 structures have been proposed to have different effects on telomerase activity on telomeres (21, 22, 24). For this reason, we assessed the difference in the ability of an oligonucleotide to bind to Cdc13-DBD depending on the topology of the G4 structure it forms (Fig. 3*B*). Notably, our results show that the folded ONG1 (parallel G4) and ONG9 (antiparallel G4) perform similarly as competitors (Fig. 3*B*), suggesting that the topology of a G4 does not have an impact on the binding of Cdc13-DBD. This might indicate that it is primarily the presence of a secondary structure on a G-overhang, not its topology, that may determine the Cdc13-binding affinity once a secondary structure is formed.

### Kinetics of G-overhang folding influence Cdc13 binding

The formation of secondary structures on telomeric ssDNA and their ability to interfere with Cdc13 binding may implicate their role on telomeres *in vivo*. In particular, our data show that G4 and G-hairpin structures have different folding kinetics, with fast-folding G4 and G-hairpin folding in a span of hours, possibly ruling out its involvement in physiological processes (Fig. 2). To elucidate this possibility, we studied whether the binding of Cdc13-DBD to the telomere-derived oligonucleotides that were allowed to fold for different time periods matched their folding kinetics. We performed EMSA experiments with unfolded, ^32^P-labeled ONG11 and competitors (ONG1 (quickly folded parallel G4) and ONG11 (slowly folded G-hairpin)) either in water (favoring the unfolded state) or in 1× K^+^ buffer (favoring the folded state) at different time points after the initiation of their folding (Fig. 4). Our experiments suggest that the G4 (ONG1) is folded after 20 min in K^+^ buffer, whereas the G-hairpin (ONG11) folding takes several hours to be completed (Fig. 2). For this reason, the ability of ONG1 and ONG11 to act as a competitor was evaluated after 20 min and 24 h after folding, as compared with the competitor in water (Fig. 4, *top*). The quantification of the free-migrating probe shows that the amount of free probe decreases with folding time for the G-hairpin–forming competitor, but it does not change for the competitor DNA folding into G4 (Fig. 4, *bottom*). These results indicate that, in agreement with our NMR folding kinetics measurements, ONG11 loses its competing ability with increasing time, suggesting an ongoing folding process, and ONG1 performs poorly as a competitor 20 min after annealing, indicating completed folding after the shorter time period. In other words, the loss of the competing ability assayed by EMSA experiments mirrors the folding kinetics of the G-hairpin and G4 structures.

To complement EMSA experiments using oligonucleotide competitors, we performed direct titrations of the Cdc13-DBD using constant concentrations of ONG1, ONG9, and ONG11 that were renatured in 1× K^+^ buffer either for 20 min (short folding) or >24 h (long folding). We hypothesized that in the case of ONG1 and ONG9, which form G4 structures quickly (Fig. 2), Cdc13-DBD would exhibit similar affinity to the probes regardless of the time of their folding. In contrast, in the case of ONG11, which is present mostly after 20 min as ssDNA, the binding was compromised when the oligonucleotide was allowed to fold during an extended time period when it reached the structure of a G-hairpin. These results are shown in Fig. S7 and support this hypothesis. The extent of the inability of Cdc13-DBD to bind to the G-hairpin is not as pronounced as in the case of the experiment presented in Fig. 4; this is probably due to interference of the phosphate group with folding of ONG11 (Fig. S8; *cf*. Note S1). However, the decreased binding of Cdc13-DBD to ONG11 in the case of the long folding is evident and reproducible, and this result supports the scenario that the length-dependent kinetics of folding of telomeric overhangs affect their ability to interact with Cdc13.

**Figure 7.**
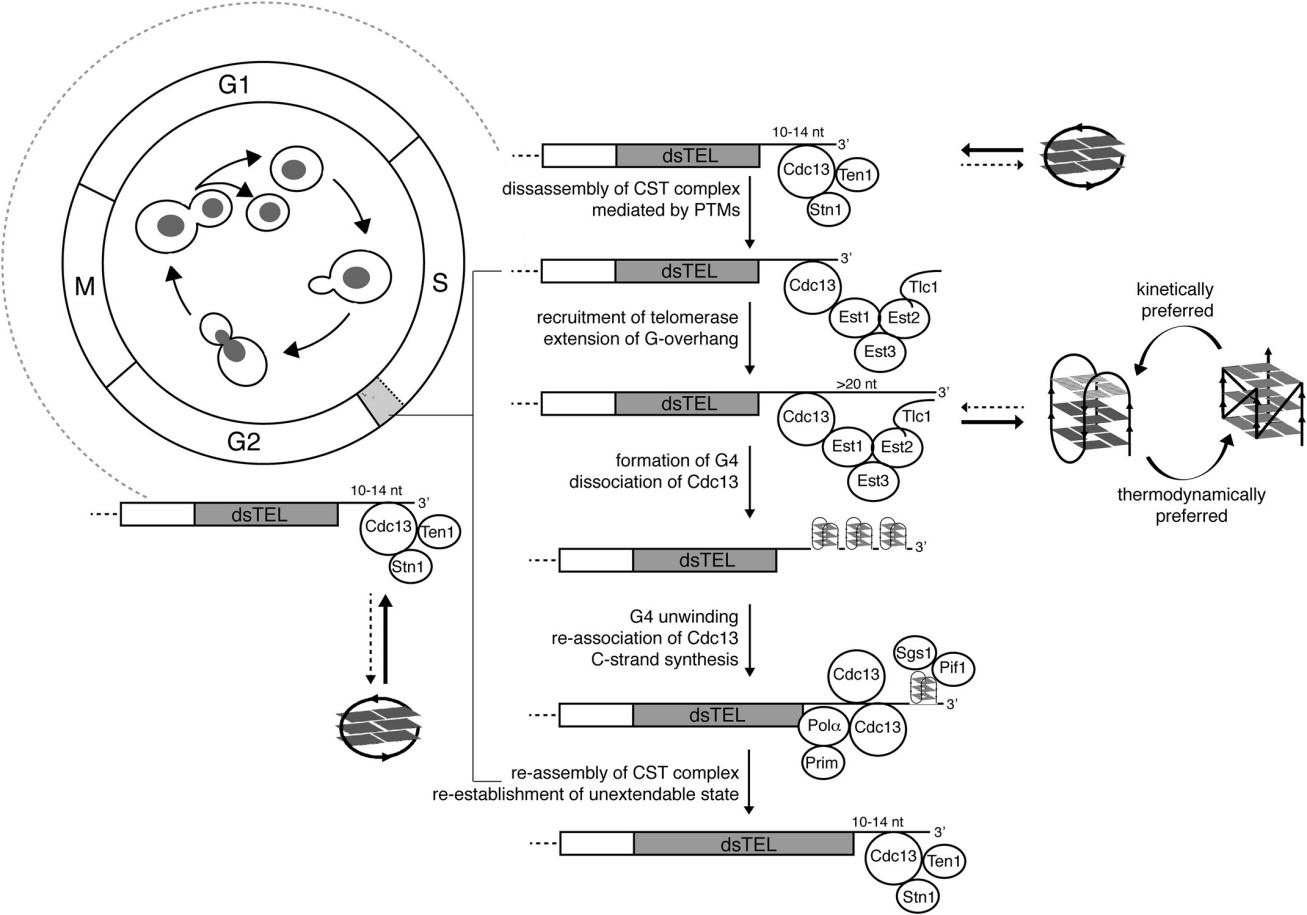
**A model summarizing the roles of non-B DNA structures formed by the G-overhang in a time-dependent manner in the maintenance of telomeres in *S. cerevisiae*.** See description under “Discussion”; for clarity, the model lacks other important players involved in G-overhang dynamics. The G4s can have distinct numbers of G-tetrads, as indicated by *different shading* of the tetrads. The *light gray box* in the cell cycle corresponds to a late S phase. *PTMs*, post-translational modifications; *Pol*α, DNA polymerase α; *Prim*, DNA primase.

EMSA-based experiments also suggested that Cdc13-DBD has a notably lower affinity toward the secondary structures formed on the telomeric DNA than to the same oligonucleotides in their single-stranded form; this implicates that the binding of Cdc13 to telomeric DNA is regulated by DNA folding kinetics. To directly rule out the possibility of interference of Cdc13 with the folding of non-B DNA structures in the course of EMSA, we acquired time-resolved NMR spectra of ONG1, ONG9, and ONG11 in the presence of equimolar amounts of Cdc13. (Note: The spectra were acquired in K^+^TD buffer). After a 100 µm solution of the ONGs was thermally denatured and cooled for ∼2 min to room temperature, it was mixed into a 100 µm solution of Cdc13-DBD. The NMR spectra of the resulting mixtures were acquired at 30, 90, and 150 min after mixing (Fig. 5). For ONG1 and ONG9, at all of the indicated time points, the overall intensities of imino signals remained essentially constant, whereas the intensity of signals in the respective spectra of ONG11 gradually increased over time (Fig. 5, *A* and *B*). Importantly, the time-dependent changes observed in the spectra of ONG1, ONG9, and ONG11 in the presence of equimolar amounts of Cdc13-DBD paralleled those observed in the absence of Cdc13-DBD (*cf*. Fig. 2*C*). Note: Compared with the NMR spectra of ONG1 acquired in K^+^S buffer (*cf*. Fig. 2), the spectra measured in K^+^TD buffer, both in the presence and absence of Cdc13-DBD, are unresolved. The unresolved character of the NMR spectrum of ONG1 in the presence/absence of Cdc13-DBD results from the increased G-quadruplex polymorphism of ONG1 in the elution (K^+^TD) buffer required for the measurements in the presence of Cdc13-DBD (see Fig. S9).

Overall the data demonstrate that Cdc13-DBD neither promotes nor hinders ONG1/ONG9/ONG11 folding. Most notably, these data directly provided information on the capacity of Cdc13-DBD to bind to G4/G-hairpin structures. In the NMR experiment, which is based on monitoring the imino signals originating exclusively from folded species, the formation of a complex between Cdc13 and G-quadruplex/G-hairpin would result in a notable increase in the line widths of the signals due to a dramatic change in the quadruplex/G-hairpin correlation time (rotational diffusion coefficient) upon complex formation. The observation of comparable linewidths between the imino signals of G-quadruplex/G-hairpin in the absence and presence of Cdc13 is evidence that neither the G-quadruplex nor the G-hairpin is capable of forming a stable complex with Cdc13 (Fig. 5*C*). The inability of Cdc13-DBD to bind G-quadruplex DNA is further corroborated by essentially identical CD spectra of both ONG1 and ONG9 acquired in the absence and presence of equimolar amount of the protein (Fig. S10).

### G4 occupancy differs through the cell cycle at the telomeric region and is in accordance with the length of the G-overhang

Immunofluorescence visualization of G4 with the BG4 antibody in human cells showed that G4 formation in the endogenous genomic region is modulated during cell cycle progression; the number of BG4 foci reached a maximum as the cells were proceeding through the S phase (57). However, due to the heterogeneous telomeric repeat motif in *S. cerevisiae*, it is unknown whether yeast telomeres produce G4 structures *in vivo*. To test whether yeast telomeres form G4 structures *in vivo,* we performed ChIP followed by qPCR. Using a specific antibody (BG4) that targets folded G4 structures, in these ChIP experiments, we pulled down G4 regions from the yeast genome. qPCR analysis allowed us to quantitatively measure G4 structure formation at telomeres (Fig. 6*A*). These data showed that at two telomeres, VI-R and VII-L G4 structures are formed. In untreated control ChIP experiments, in which no BG4 antibody was added, no binding was observed. As an additional control, we treated the cells with the highly specific G-quadruplex ligand, Phen-DC_3_ (58). After treatment, an increase in telomeric G4 was detectable after ChIP and qPCR. Next, we tested whether G4 formation is cell cycle–regulated and whether this regulation is in agreement with length changes of the telomere overhang. To address this, *S. cerevisiae* cells were arrested in the G_1_ phase with the mating pheromone α factor and released into the cell cycle with varying concentrations of hydroxyurea (HU) to segment the S phase and the anti-microtubule assembly agent, nocodazole, to arrest the cells in G_2_ phase (Fig. 6*B*). Next, we quantitatively measured G4 occupancy at the VII-L telomeric region by ChIP and qPCR. Cell cycle progression was controlled by FACS. The lowest level of telomeric G4 enrichment was observed in the early S phase (250 mm HU), with a slight increase in the mid-S phase (150 mm HU) and the highest level (2–3-fold) of accumulation in the late S phase (75 mm HU) (Fig. 6*C*). As pointed out before, Cdc13 is essential for capping the ssDNA at telomeric ends, and it was demonstrated that G4 DNA might have a capping role when the natural capping is compromised (42). We speculated that more G4 structures should be present at the telomeric region if the Cdc13-dependent capping mechanism is impaired. For this, we used the temperature sensitive *cdc13-1* mutant. In this mutant, telomeres are uncapped if cells are grown under nonpermissive temperature. This uncapping leads to long tracts of guanine-rich single-stranded telomeric DNA (51). We reasoned that the long G-overhang in *cdc13-1* mutant telomeres might form G4 DNA at elevated temperatures. To test this, we did BG4 ChIP at permissive (23 °C, normal telomeres) and nonpermissive temperatures (37 °C, uncapped telomeres) and checked the telomeric G4 levels. As expected, we observed higher telomeric G4 enrichment in *cdc13-1* strain at nonpermissive temperature (37 °C) compared with permissive temperature (23 °C), as well as WT (Fig. 6*D*). Notably, these data support *in vitro* observations that G4 structures preferentially form during the time when long telomeric overhangs are present at the chromosome ends (46, 47).

## Discussion

It is generally recognized that the major functions of telomeres are mediated by telomere-associated proteins. During most of the cell cycle, Cdc13 associates with proteins Stn1 and Ten1, thus forming the CST complex (59–61) that protects the overhang from exonucleases and prevents access to telomerase (62, 63). Orchestrated by a number of post-translational modifications, the association of Cdc13 with Stn1 and Ten1 is exchanged for the association with the telomerase subunit Est1 during the S phase, thus ensuring the enzyme recruitment to telomeres (33, 50, 64–66). In the late S/G_2_ phase, telomere extension is followed by the restoration of CST at the G-overhangs, making them inaccessible to telomerase until the next late S phase (67, 68).

In addition to the regulation of Cdc13 by post-translational modifications and protein-protein interactions, the events taking place at telomeres are affected by the non-B DNA structures adopted by the G-overhang; however, their contribution to telomere function is not yet understood. Here, we showed that the oligonucleotides emulating an extended telomeric DNA overhang that is characteristic of the late S phase of the *S. cerevisiae* cell cycle can form two typologically distinct, antiparallel and parallel, intramolecular G4 structures. Although our data demonstrate that a concurrent formation of both parallel and antiparallel G4 structures is possible in a specific sequence context, the formation of an intramolecular antiparallel G4 appears kinetically favored over the formation of an intramolecular parallel G4. Our observation of comparable capacities of parallel and antiparallel G4 structures to displace Cdc13 from the complex with ssDNA suggests that the biological function of G4 is either not connected with a specific G4 folding topology or is associated with the kinetically preferred antiparallel G4.

Previously, it was shown that Cdc13 and Cdc13-DBD can bind the tetramolecular parallel G4 with a similar affinity as the ssDNA (unfolded) form and that the binding is accompanied by a partial denaturation of the tetramolecular G4 structure, stabilized by Na^+^ cations (41). In contrast to these observations, our data indicate that binding of both intramolecular parallel and antiparallel telomeric G4s bind to Cdc13-DBD is considerably weaker compared with its binding to telomeric DNA in a single-stranded form (Figs. 4 and 5). Moreover, in contrast to the published results (41), our data indicate that Cdc13-DBD does not induce denaturation of the intramolecular G4 structure (Fig. 5 and Fig. S10). We assume that these differences may reflect the different nature of the studied G4 structures (intermolecular *versus* intramolecular, Na^+^- *versus* K^+^-stabilized). In fact, different affinity and rate of unfolding were reported for Na^+^ *versus* K^+^ G4 interacting with human POT1-TPP1 telomeric protein complex (69). However, our experiments describe the behavior of sequences derived from the most represented repeat in yeast telomeres (48) and may thus more closely reflect the situation *in vivo*.

The limitation of our study is the use of Cdc13-DBD instead of a Cdc13 full-length protein. Although we resorted to this approach due to technical problems with the full-length protein purification, we acknowledge that other domains of Cdc13 can also contribute to its interaction with G4 or G-hairpin structures. However, the affinity toward the telomeric ssDNA motif is similar for the two proteins (54), as is also the previously observed affinity toward the interaction with tetramolecular G4 structure (41), suggesting that their behavior may be similar also for the substrates we studied. The binding of several different substrates can be mediated by the ability of DBD to interact with different secondary structures in a different manner, as is the case for the DBD of the yeast telomeric protein Rap1 and its binding to ssDNA *versus* G4 structure (70).

Our data suggest that Cdc13 preferably binds to G-rich telomeric ssDNA, whereas the formation of intramolecular G4 impairs its binding, regardless of the G4 folding topology. This observation is consistent with the evolution-based argument suggesting that the topology of non-B DNA motifs formed in a G-overhang should be neutral with respect to their function (17). To assess the physiological relevance of non-B DNA structures at long and short telomeric overhangs, we compared the capacity of Cdc13 to bind to unfolded, G-hairpin, and G4 telomeric DNA. Similar to G4, the G-hairpin displayed a diminished capacity to form a stable complex with Cdc13 compared with that of unfolded DNA (Figs. 4 and 5). The binding of Cdc13-DBD observed by EMSA reflects the folding kinetics of different secondary structures, potentially hinting at their physiological roles. The typical time required to complete the cell cycle for *S. cerevisiae* is ∼90 min, with the S phase spanning <10 min. Any non-B DNA structure to take part in related biological processes must be formed with a relevant time window (*i.e.* ≪10 min for a G4 within a long telomeric overhang and ≪80 min for a G-hairpin within a short telomeric overhang. For G4 structures, which are known to fold on the time scale of seconds/minutes (71) (Fig. 2*C*), this condition is fulfilled; for G-hairpins, it is not, as the time required to completely fold this structure exceeds several hours (Fig. 2*C*).

Within the timeframe of a cell cycle, the short G-overhang sequence displaying a G-hairpin–forming potential predominantly exists in its unfolded form, which, in contrast to both G4 and G-hairpin forms (Figs. 4 and 5), has a high affinity to Cdc13 (Fig. 4). We suggest that non-B DNA structure formation serves as a kinetically controlled switch to regulate the binding of Cdc13 to telomeric G-overhangs (Fig. 7). Whereas the situation *in vivo* may be more complicated because of other proteins interfering with non-B DNA structure formation, our NMR measurements in cell lysates support the possibility that the folding kinetics may be similar to the *in vitro* situation (Fig. S2). To the best of our knowledge, these data show for the first time how the secondary structures forming at a G-rich overhang may play a physiological role in yeast telomeres. Based on our data, we propose a model summarizing how rapid G4 formation on the elongating 3′ overhang participates in the telomerase-driven extension of telomeres to fine-tune the regulation of telomerase dissociation. During most of the cell cycle, a short G-overhang is refractory to forming secondary structures and bound by the CST complex that prevents telomerase recruitment. During the S phase, because of post-translational modifications of Cdc13 (33, 50, 64–66), Cdc13 mediates the recruitment of telomerase to telomeres. After elongation by telomerase, the elongated overhangs are prone to form fast-folding G4 structures. The formation of G4s at a G-overhang may be followed by the dissociation of Cdc13 due to its low affinity toward G4 structures. As has been shown previously for stabilized G4 structures at telomeres in the absence of Cdc13, a G4 may transiently act as telomere cap in the absence of Cdc13 (42). After the following unfolding of G4 structures by a concerted action of telomerase (22) and helicases (30, 72), the G-overhang may be bound again by multiple Cdc13 molecules. As a result of these events, there is an increase in the accumulation of telomerase, G4, and Cdc13 at telomeres during the late S phase (Fig. 6). Subsequently, Cdc13-containing ends serve as substrates for C-strand fill-in synthesis machinery (73), thus restoring short, CST-bound G-overhangs that are refractory to telomerase binding and refractory to quickly form a secondary structure (Fig. 7). It is probable that all of these steps are subject to additional levels of regulation, such as the action of various nucleases/helicases and post-translational modifications that may affect the affinity of Cdc13 not only for its protein partners but also for various structures formed by a G-overhang. In addition, currently, it is not clear how the length and structure of individual G-overhangs affect the telomerase activity. Furthermore, our data show the sum of different telomere lengths, and we do not know whether G4s observed by BG4 are only forming at the overhang or if they also form at telomeric dsDNA regions. These and other questions will need to be investigated in detail in future studies.

It is interesting, however, to address possible parallels between yeast and human telomeres based on our observations. Although the length of the human telomeric overhang remains sufficient to form G4 structure throughout the cell cycle, it has been shown that POT1, the main human ssDNA-binding telomeric protein, alone or in complex with TPP1, is capable of unwinding G4 structures *in vitro*, thus enabling telomere extension by telomerase (25, 69). Moreover, the human CST complex (homolog of its yeast counterpart) has also been described to bind and unfold G4 structures, contributing to removing barriers for replication fork progression (74, 75). These findings suggest an evolutionarily conserved role for G4 structures in telomerase regulation and for CST complex as an interacting partner for G-quadruplexes from yeast to humans.

## Conclusions

Formation of non-B DNA structures in the G-overhang is considered to be an epigenetic hallmark of telomeric DNA. In *S. cerevisiae*, these structures involve G-hairpins and topologically distinct classes of intramolecular G4 structures. Here, we propose that the time-dependent formation of non-B DNA structures plays an active regulatory role in telomere maintenance. This study shows for the first time how different lengths of G-overhangs may influence the formation of non-B DNA structures and suggests a possible physiological role for G4s in G-overhangs. Our model connects the time-controlled formation of non-B DNA structures with cell cycle–regulated lengths of G-overhangs and binding by Cdc13. Altogether, our data support a notion of non-B DNA structures in G-overhangs as active players in telomere maintenance and highlight the important role of the time-dependent process of non-B DNA structure formation.

## Experimental procedures

### Samples for spectroscopic experiments

DNA oligonucleotides (Table 1) were purchased from Sigma–Aldrich and were dissolved in H_2_O to yield 1 mm aqueous stock solutions. The oligonucleotide folding was performed by heating the stock solutions to 95 °C for 10 min, followed by cooling them to room temperature. The precise oligonucleotide concentrations were determined from the UV absorbance measured on a NanoDrop 2000c (Thermo Fisher Scientific). The stock solutions were used for the sample preparation for both NMR and CD analyses. The CD and NMR spectra were measured in K^+^S buffer (20 mm potassium phosphate, pH 7.0, 135 mm KCl) unless stated otherwise.

### End-labeled oligonucleotide used as an EMSA probe

The oligonucleotide ONG11 (Microsynth, Table 1) was labeled on its 5′ end by T4 polynucleotide kinase (Thermo Fisher Scientific) according to the manufacturer's instructions. The unincorporated [γ-^32^P]ATP was removed by a Probe Quant G-25 Micro Column (GE Healthcare) equilibrated with water; the probe was stored at 4 °C and boiled immediately prior to each experiment.

### Unlabeled oligonucleotides used as EMSA competitors

DNA oligonucleotides were purchased from Sigma–Aldrich (ONG9) or Microsynth (all remaining oligonucleotides) (Table 1). The oligonucleotide working solutions (50 µm oligonucleotide) in either water or 1× K^+^ buffer (20 mm potassium phosphate, pH 7.0, 135 mm KCl, 9% (v/v) glycerol) were prepared by diluting the oligonucleotide stock solutions (100 or 200 µm oligonucleotides prepared in water) with the appropriate amount of water and/or 4× K^+^ buffer. The folding was performed as described above. All oligonucleotides were diluted to the working concentrations in water and/or 4× K^+^ buffer immediately prior to the experiments.

### Purification of Cdc13-DBD

The coding sequence for the Cdc13-DBD (residues 497–694) was amplified from genomic DNA of the *S. cerevisiae* strain SCY325 (*MAT*α, *ade2-1, his3-11,15, leu2-3,112, trp1-1, ura3-1,* and *can1*) by PCR using the primers Cdc13-DBD-F (5′-phospho-AGGATGAGCAAAATGGCAAGGAA-3′) and Cdc13-DBD-R (5′-phospho-CGCGAGATGAGAACCGTTTCTAT-3′). The PCR fragment was ligated into the pGEX-6T-2 vector (GE Healthcare) linearized by SmaI and dephosphorylated using alkaline phosphatase. The construct sequence was verified by restriction digestion and sequencing. The expression of the Cdc13-DBD–encoding gene was induced in BL21(DE3) *E. coli* by adding 1 mm isopropyl 1-thio-β-d-galactopyranoside to a mid-log phase culture and subsequently incubating for 4 h at 22 °C and 225 rpm. The cells were washed once with ice-cold PBS, collected, and stored at −80 °C. The thawed cells were resuspended in 1× K^+^ buffer containing 1 mg/ml lysozyme (Sigma–Aldrich), 1× cOmplete Protease Inhibitor mixture (Roche Applied Science), 10 μg/ml leupeptin (Applichem), 1 mm DTT (Thermo Fisher Scientific), 10 mm MgCl_2_ (Sigma–Aldrich), 30 μg/ml RNase A (Invitrogen), and 10 units/μl DNase I (Sigma–Aldrich). The suspension was incubated on ice for 15 min followed by sonication (6 × 20 s, 30% amplitude, model 120 Sonic Dismembrator (Fisher)). Triton X-100 (Sigma–Aldrich) was added to a final concentration of 0.25% (v/v) followed by three additional sonication pulses. After incubating for 5 min on ice, the suspension was centrifuged for 20 min at 10,000 × *g* and 4 °C in an SS-34 rotor (Sorvall). The resulting supernatant was mixed with 0.5-ml GSH-agarose beads (Sigma–Aldrich) equilibrated with 1× K^+^ buffer containing 0.25% Triton X-100 and 1 mm DTT (K^+^TD buffer) with 1× cOmplete Protease Inhibitor mixture (Roche Applied Science) and incubated end-over-end for 3 h at 4 °C. The beads were then washed three times with K^+^TD buffer containing 1× cOmplete Protease Inhibitor mixture (Roche Applied Science) and four times with K^+^TD without the protease inhibitors. The beads were resuspended in 0.7 ml of K^+^TD buffer without protease inhibitors containing the HRV 3 °C protease and incubated end-over-end for 3 h at 4 °C. The flow-through was collected as the elution fraction. The protein concentration was determined by a Bradford assay (76), and the samples were stored at −80 °C.

### Yeast lysate preparation

A 500-ml culture of *S. cerevisiae* BY4741 (*MATa*, *his3*Δ, *leu2*Δ, *met15*Δ, *ura3*Δ) was grown overnight at 30 °C and 180 rpm to a density of 3 × 10^7^ cells/ml. Cells were harvested, resuspended in 1× K^+^ buffer, and disrupted by FastPrep-24 (MP Biomedicals). Then 1× K^+^ buffer was added to a final volume of 2 ml, and the extract was sonicated for 60 s at 30% amplitude with the model 120 Sonic Dismembrator (Fisher).

### EMSA

All DNA-binding reactions were performed in 1× K^+^ buffer. After the addition of the labeled probe, the reactions were incubated for 10 min at room temperature and then loaded on a 6% polyacrylamide gel in 0.5× TBE buffer (45 mm Tris borate, 1 mm EDTA, pH 8.0). The electrophoresis was performed in a mini-PROTEAN Tetra Cell R (Bio-Rad) for 18 min at 10 mA/gel. The gels were then fixed with 10% (v/v) methanol and 10% (v/v) acetic acid, vacuum-dried, exposed to a phosphor screen, and visualized using Personal Molecular Imager FX (Bio-Rad).

### CD spectroscopy

CD spectra were measured using a JASCO J-815 spectrometer in 1-mm path-length quartz cells placed in a Peltier holder. CD signals are expressed as the difference in the molar absorption of the left- and right-handed circularly polarized light. The molarity was related to the DNA strands. Spectra were acquired at a rate of 100 nm/min and averaged from three measurements.

### Nondenaturing PAGE

Nondenaturing PAGE was performed in a temperature-controlled electrophoresis cell (PROTEAN II xi, Bio-Rad) submerged in a cooling system. The gels (16%, 29:1 acrylamide/bisacrylamide) in 16 20-cm glass cassettes were electrophoresed for 22 h at 40 V and 7 °C in potassium phosphate buffer. The gels were stained with Stains-All (Sigma–Aldrich).

### NMR spectroscopy

The 1D ^1^H spectra of the DNA samples were acquired at 700 MHz using a Bruker Avance III NMR spectrometer equipped with a triple resonance room temperature probe using the zggpw5, p3919 (standard Bruker library) or 1-1 echo pulse sequences (77). All of the spectra were measured in a water solution (90% H_2_O, 10% D_2_O) at 25 °C unless stated otherwise and referenced to the signal of residual H_2_O. To assess the folding kinetics from the NMR spectra, the intensity of signals in the imino region (exchangeable protons) normalized to the intensity of signals in the aromatic region (nonexchangeable protons) of the same NMR spectrum was plotted as a function of time.

### G4 ChIP

G4 ChIP was performed using the G4 structure-specific antibody (BG4), as described previously (78, 79). Briefly, the BG4 recombinant antibody was prepared using the expression vector pSANG10-3F-BG4 (a gift from Shankar Balasubramanian (Addgene plasmid 55756; RRID:Addgene_55756 (57)) in BL21(DE3) *E. coli.* The purified BG4 solution was concentrated using an Amicon Ultra-15 centrifugal filter unit (Millipore, catalog no. UFC9010). For the ChIP experiment, *S. cerevisiae* strains were grown and cross-linked with 1% (v/v) formaldehyde. Cross-linked samples were immunoprecipitated with anti-FLAG M2 magnetic beads (Sigma–Aldrich) in the presence or absence of the BG4 antibody and 10 μm Phen-DC_3_ to determine the enrichment. The enrichment of telomeric DNA was confirmed by qPCR with primers Tel VIIL_for (TGATATGTGTTACGCAGAATAC-3′), Tel_VIIL_rev (TGAGAAGCACCGCAATG-3′), Tel VI-R for (ATCATTGAGGATCTATAATC-3′), and Tel VI-R rev (CTTCACTCCATTGCG-3′) that are specific for the VII-L and VI-R telomeric regions. To obtain the fraction of recovery (percentage of input), the results (*Cq* values) were normalized to the relative input. All data are depicted as the mean ± S.E., *n* = 3. *Error bars* indicate the SEM.

### Cell cycle analysis

Cell cycle synchrony experiments were performed as described previously (80). Briefly, *S. cerevisiae* cells (W303; *MATa*, *leu2-3,112*, *trp1-1*, c*an1-100*, *ura3-1*, *ade2-1*, *his3-11,15, bar1*Δ∷KanMX) (81) were arrested in the G_1_ phase with the α factor at a concentration of 5 μg/ml for 3 h. The cells were checked under the microscope for shmoo formation. G_1_-arrested cells were released into the cell cycle in the presence of different concentrations of HU for early (250 mm), mid (150 mm), and late (75 mm) S-phase arrest and 15 μg/ml nocodazole for G_2_ arrest. Samples were taken for DNA content analysis by a BD FACS Canto II. G4 ChIP was performed with cell cycle–arrested cells as described above.

## Data availability

All data are contained within the article.

## Supplementary Material

Supporting Information
