# Supplementary material for: Role of folding kinetics of secondary structures in telomeric G-overhangs in the regulation of telomere maintenance in Saccharomyces cerevisiae
Source: J Biol Chem. 2020 May 8;295(27):8958–71. doi: 10.1074/jbc.RA120.012914 (PMC7335780; doi:10.1074/jbc.RA120.012914)
Supplement: Supporting Information [file supp_295_27_8958__index.html]

Role of folding kinetics of secondary structures in telomeric G-overhangs in the regulation of telomere maintenance in Saccharomyces cerevisiae — Length-dependent folding kinetics of telomeric overhangs — Role of folding kinetics of secondary structures in telomeric G-overhangs in the regulation of telomere maintenance in Saccharomyces cerevisiae — Length-dependent folding kinetics of telomeric overhangs — Supporting Information 

# Role of folding kinetics of secondary structures in telomeric G-overhangs in the regulation of telomere maintenance in *Saccharomyces cerevisiae*

## Supporting Information

- Supporting Information (to be published online) - Supplementary Notes and Figures S1-S10
