# Supplementary material for: Role of folding kinetics of secondary structures in telomeric G-overhangs in the regulation of telomere maintenance in Saccharomyces cerevisiae
Source: J Biol Chem. 2020 May 8;295(27):8958–71. doi: 10.1074/jbc.RA120.012914 (PMC7335780; doi:10.1074/jbc.RA120.012914)
Supplement: Supporting Information [file supp_RA120.012914_158633_2_supp_526148_q9y8wj.pdf]

Juríková *et al.*

SUPPLEMENTARY INFORMATION

Supplementary Note S1: In the direct binding assay (Figure S7), the binding between 5'-phosphate terminally modified ONG11 with Cdc13-DBD is assessed. The impact of terminal phosphate on ONG11 folding (cf. Figure S8) directly translates into apparent binding affinity of the ONG with respect to Cdc13-DBD. Note: In contrast, in the indirect (competitor) based assay (Figure 4), the 5'-phosphate terminally modified ONG11 is pre-bound to Cdc13-DBD in its unfolded form and it is an impact of folding of unmodified ONG11 on Cdc13-DBD binding, which is assayed.

List of Supplemementary Figures

Supplementary Figure S1. (A) Native PAGE of ONG1-11. All studied constructs migrate on native PAGE as mono-molecular species. Two bands visible for ONG4 correspond to two distinct intra-molecular (G4) species (cf. (B)) with notably different hydrodynamic behavior. For other ONGs, which also form polymorphic mixtures, these differences appear less pronounced and the resolution of native PAGE is insufficient to discriminate among them. (B) Time evolution of imino region of 1D ^{1}H NMR spectrum of ONG4 supports existence of at least two G-quadruplex species. Major and minor G-quadruplex species is highlighted by red and blue lines, respectively.

Supplementary Figure S2. Intracellular factors do not promote G-hairpin folding. Time evolution of imino region of 1D ^{1}H NMR spectra from 50 μM ONG1 and ONG11 in cellular lysate. The spectra were measured using 1-1 echo pulse sequence at 25 ºC. Note: In contrast to the experiment presented in **Figure 5**, where behavior of ONG1 and ONG11 is monitored in the presence of equimolar amount of Cdc13-DBD, the DNA concentrations are expected to be in excess with respect to the individual endogenously present proteins in the cell extract based experiment.

Supplementary Figure S3. Purification of Cdc13-DBD. The expected size (23.34 kDa) and purity of the isolated Cdc13-DBD protein were checked by 12% SDS-PAGE. E1, E2: elution fractions.

Supplementary Figure S4. EMSA experiments confirm the previously observed binding properties of Cdc13. Left: the binding of [^{32}P]-labelled ONG11 by Cdc13-DBD was assayed in direct EMSA with increasing protein concentration. Right: ONG11 and Cdc13-NC were used as unlabelled competitors in an EMSA with [^{32}P]-labelled ONG11 to confirm the binding preference of Cdc13-DBD. ONG11 is a previously described binding motif of Cdc13, whereas Cdc13-NC (5'-TGTGGGTGTG-3') has been previously used as a negative control for Cdc13 binding (1, 2).

Supplementary Figure S5. Formation of non-B DNA secondary structures requires presence of K^+ ions. A) Imino regions of 1D ^{1}H NMR spectra of ONG1, ONG9, and ONG11 acquired in miliQ (MQ) H_2O as a function of time. Unresolved signals at ~ 10.6 ppm in the NMR spectrum of ONG1 acquired in MQ H_2O corresponds to imino protons involved in G-G base-pairs in pre-folded G4/G-hairpin intermediate(s) (3). B) Comparison of NMR spectra acquired in potassium-based (K^+S) buffer (black) and in MQ H_2O (red) at t = 24 hours after annealing. C) Comparison of CD spectra in K^+S buffer 24 hours after annealing (black) and CD spectra acquired in MQ H_2O 10 minutes (blue) and 24 hours after annealing (red). The spectra were acquired using zggpw5 pulse sequence in K^+S buffer at 25 ºC.

Supplementary Figure S6. **Titration of ONG11 competitor in its unfolded and folded (G-hairpin) state illustrates a preference of Cdc13-DBD for single-stranded DNA.**

Supplementary Figure S7. Length-dependent kinetics of folding of telomeric overhangs affect their ability to interact with Cdc13-DBD. The corresponding oligonucleotides were labelled at 5' end, diluted in K^+ buffer to the concentration of 15 nM, incubated at 100 ºC for 5 min and cooled down to room temperature for either 20 min (short folding) or >24 hours (long folding). The "long

folding“ probes were kept at 4 ºC, the "short folding“ oligonucleotides were prepared immediately prior each experiment. After incubation with the indicated concentrations of Cdc13-DBD, the DNA-protein complexes were resolved using 6% polyacrylamide gel as described in Materials and Methods. The graph visualizes quantification of shifted probe, mean ± s.d., n = 2 (ONG1, ONG9) and n = 3 (ONG11) independent replicas. *$p<0.05$.

Supplementary Figure S8. **5'-terminal phosphate moiety slows down G-hairpin folding**: Imino region of ^{1}H 1D NMR spectra of ONG11 with 5'-terminal -PO_3H (A) and 5'-terminal -OH (B) acquired as a function of time (indicated). Imino regions of the spectra reporting on a population of the folded species (G-hairpin) are highlighted (red box). (C) Overlay of imino region of the ^{1}H 1D NMR spectra of ONG11 with 5'-terminal -PO_3H (black) and 5'-terminal -OH (red). The spectra were acquired using p3919 pulse sequence (standard Bruker library) in K^{+}S buffer at 25 ºC.

Supplementary Figure S9. Impact of buffer composition on appearance of NMR spectra. Imino regions of ^{1}H 1D NMR spectra of ONG11 acquired in K^{+}S (A) and K^{+}TD (B) buffer. The spectra in A) and B) were acquired using zggpw5 and 1-1 echo pulse sequence, respectively, at 25 ºC.

Supplementary Figure S10. Cdc13 does not induce denaturation of G4 structure. Left: CD spectra of ONG1. Right: CD spectra of ONG9. Control samples with DNA alone are indicated in red. Samples with Cdc13-DBD:ONG in 1:1 ratio were recorded immediately after Cdc13-DBD addition (blue) and after 30 min (black).

References

1. Hughes,T.R., Weilbaecher,R.G., Walterscheid,M. and Lundblad,V. (2000) Identification of the single-strand telomeric DNA binding domain of the *Saccharomyces cerevisiae* Cdc13 protein. *Proc. Natl. Acad. Sci. USA*, **97**, 6457–6462.
2. Anderson,E.M., Halsey,W.A. and Wuttke,D.S. (2002) Delineation of the high-affinity single-stranded telomeric DNA-binding domain of *Saccharomyces cerevisiae* Cdc13. *Nucleic Acids Res.*, **30**, 4305–4313.
3. Čeru, S., Šket, P., Prislan, I., Lah, J. and Plavec, J. (2014) A new pathway of DNA G-quadruplex formation. *Angew. Chem. Int. Ed. Engl.*, **53**, 4881–4884.

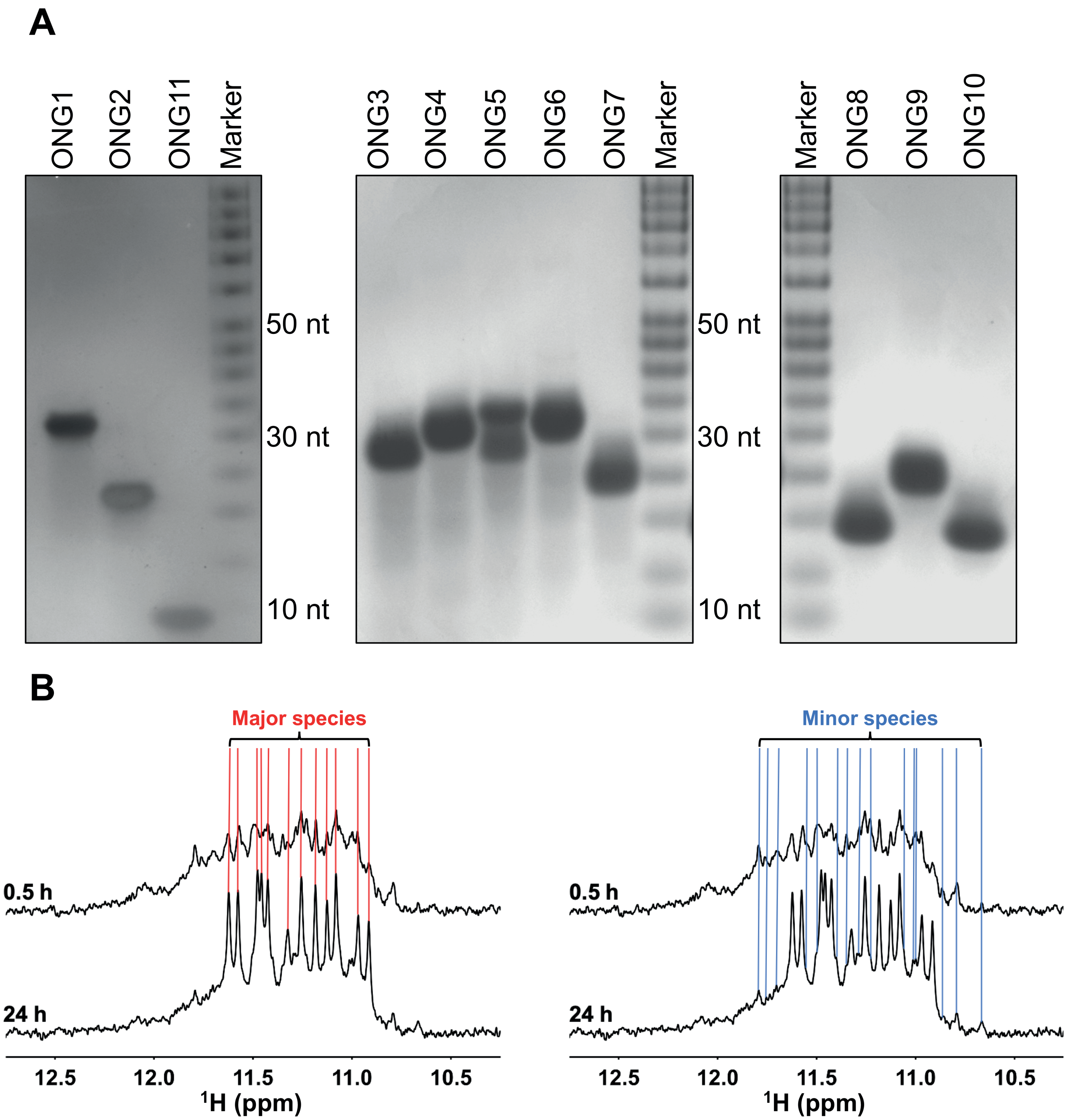

Supplementary Figure S1. (A) Native PAGE of ONG1-11. All studied constructs migrate on native PAGE as mono-molecular species. Two bands visible for ONG4 correspond to two distinct intra-molecular (G4) species (cf. (B)) with notably different hydrodynamic behavior. For other ONGs, which also forms polymorphic mixtures, these differences appear less pronounced and the resolution of native PAGE is insufficient to discriminate among them. (B) Time evolution of imino region of 1D ^{1}H NMR spectrum of ONG4 supports existence of at least two G-quadruplex species. Major and minor G-quadruplex species is highlighted by red and blue lines, respectively.

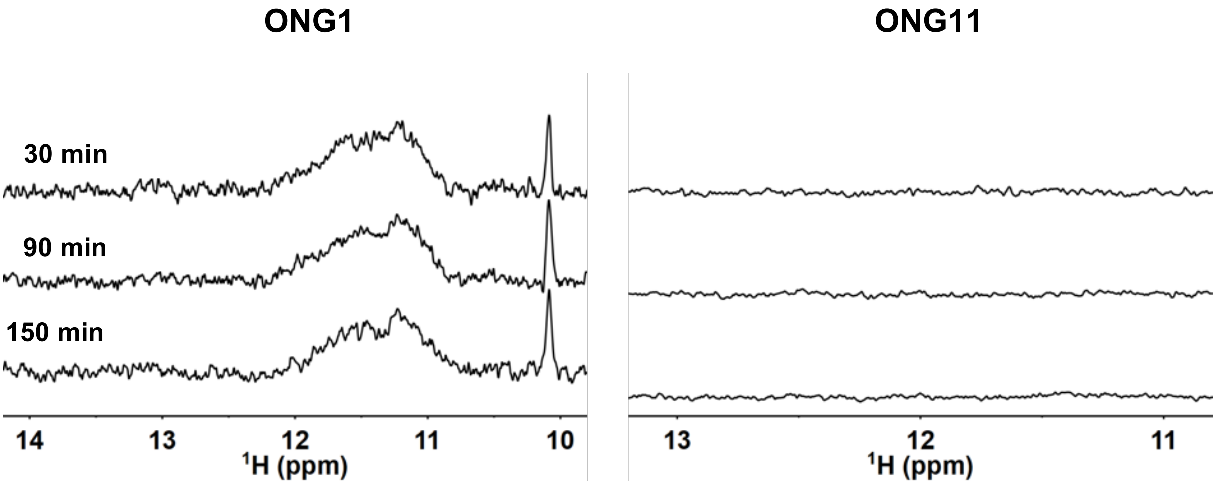

Supplementary Figure S2. Intracellular factors do not promote G-hairpin folding. Time evolution of imino region of 1D ^{1}H NMR spectra from 50 mM ONG1 and ONG11 in cellular lysate. The spectra were measured using 1-1 echo pulse sequence at 25 °C. Note: In contrast to the experiment presented in **Figure 5**, where behavior of ONG1 and ONG11 is monitored in the presence of equimolar amount of Cdc13-DBD, the DNA concentrations are expected to be in excess with respect to the individual endogenously present proteins in the cell extract based experiment.

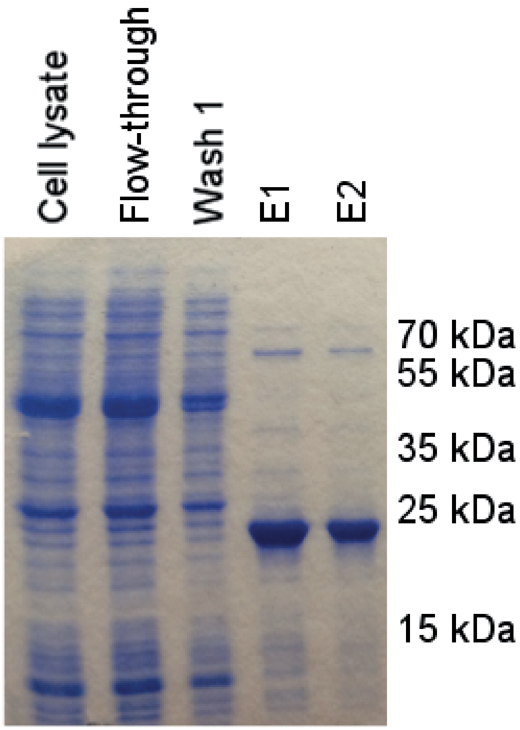

Supplementary Figure S3. Purification of Cdc13-DBD.
The expected size (23.34 kDa) and purity of the isolated Cdc13-DBD protein was checked by 12% SDS-PAGE.
E1, E2: elution fractions.

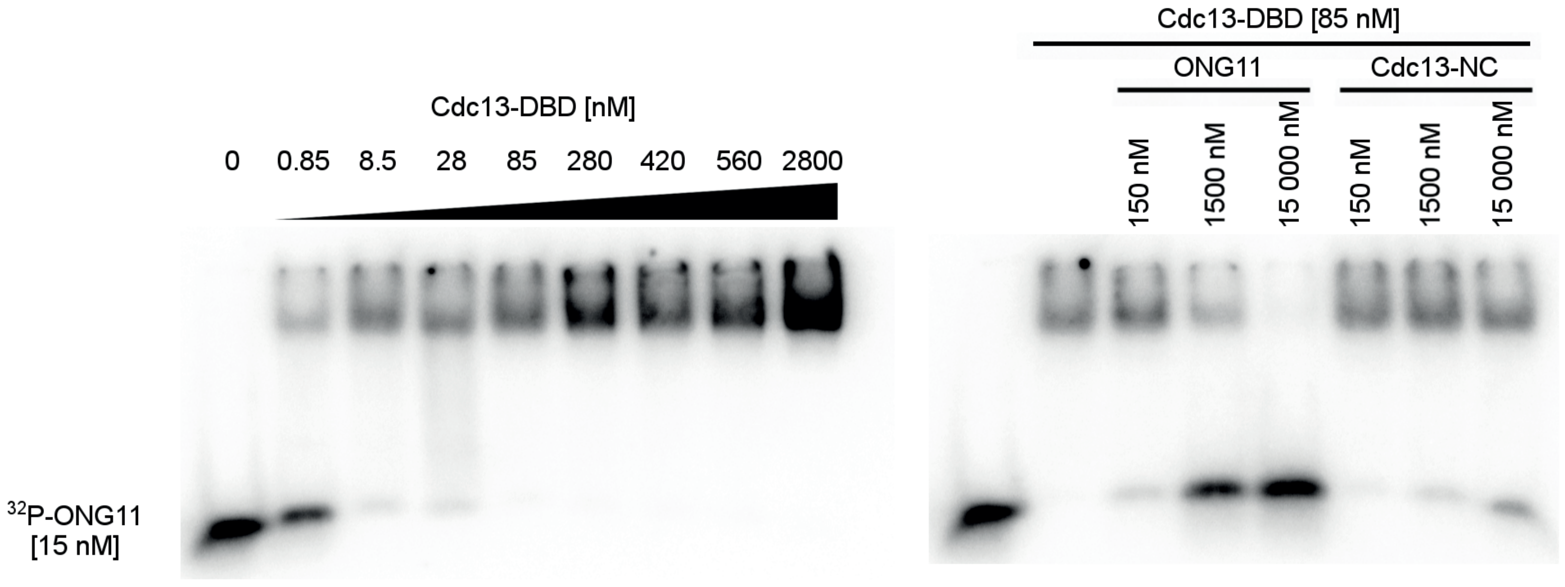

Supplementary Figure S4. EMSA experiments confirm the previously observed binding properties of Cdc13. **Left:** the binding of [^{32}P]-labelled ONG11 by Cdc13-DBD was assayed in direct EMSA with increasing protein concentration. **Right:** ONG11 and Cdc13-NC were used as unlabelled competitors in an EMSA with [^{32}P]-labelled ONG11 to confirm the binding preference of Cdc13-DBD. ONG11 is a previously described binding motif of Cdc13, whereas Cdc13-NC (5'-TGTGGGTGTG-3') has been previously used as a negative control for Cdc13 binding (1, 2).

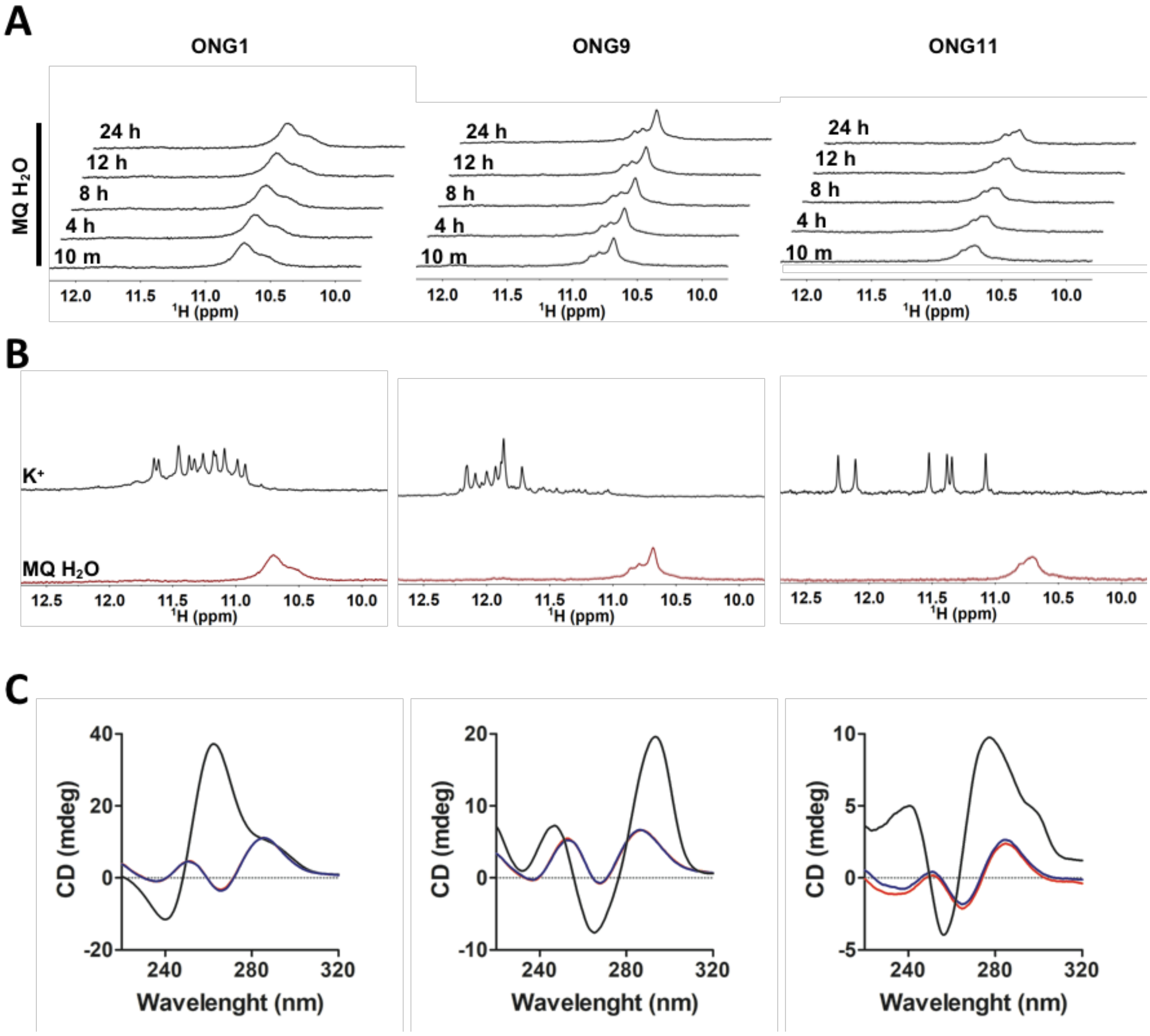

Supplementary Figure S5. Formation of non-B DNA secondary structures requires presence of K^+ ions. A) Imino regions of 1D ^{1}H NMR spectra of ONG1, ONG9, and ONG11 acquired in miliQ (MQ) H_2O as a function of time. Unresolved signals at ~ 10.6 ppm in the NMR spectrum of ONG1 acquired in MQ H_2O corresponds to imino protons involved in G-G base-pairs in pre-folded G4/G-hairpin intermediate(s) (3). B) Comparison of NMR spectra acquired in potassium-based (K^+S) buffer (black) and in MQ H_2O (red) at t = 24 hours after annealing. C) Comparison of CD spectra in K^+S buffer 24 hours after annealing (black) and CD spectra acquired in MQ H_2O 10 minutes (blue) and 24 hours after annealing (red). The spectra were acquired using zggpw5 pulse sequence in K^+S buffer at 25 ºC.

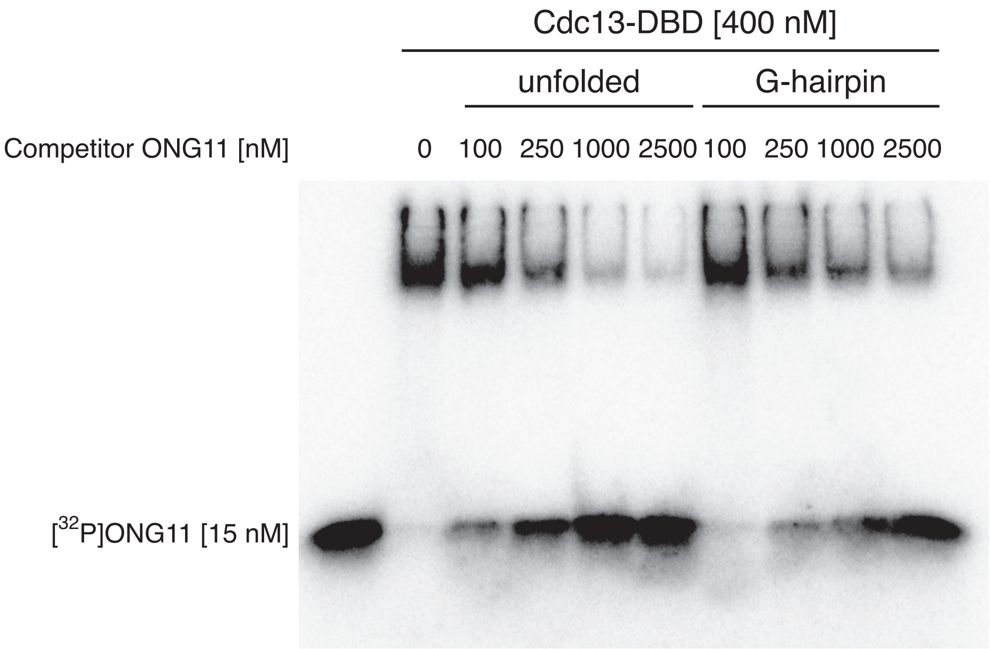

Supplementary Figure S6. Titration of ONG11 competitor in its unfolded and folded (G-hairpin) state demonstrates a preference of Cdc13-DBD for single-stranded DNA.

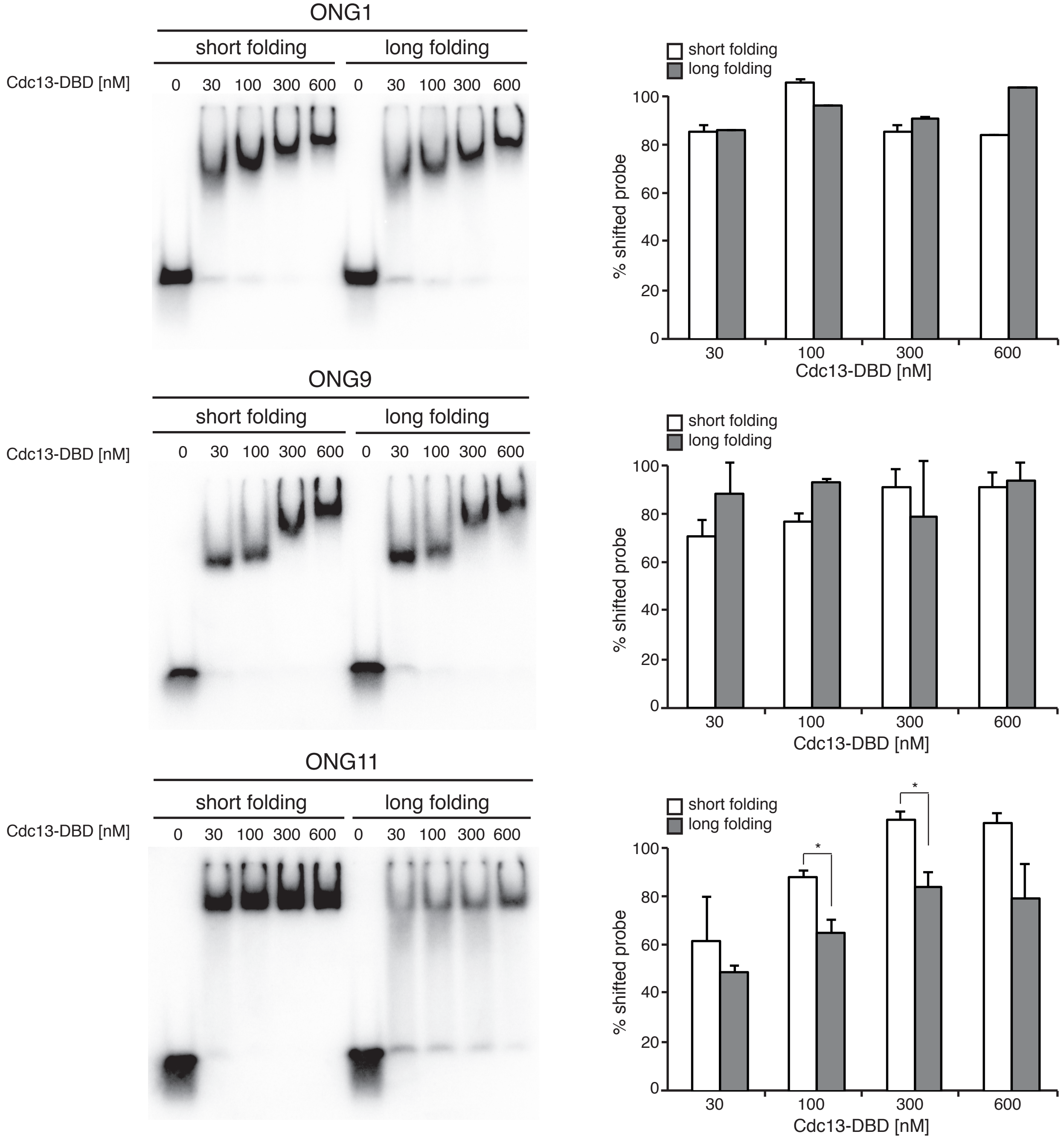

Supplementary Figure S7. The corresponding oligonucleotides were labelled at 5' end, diluted in K^+ buffer to the concentration of 15 nM, incubated at 100 ^oC for 5 min and cooled down to room temperature for either 20 min (short folding) or >24 hours (long folding). The “long folding“ probes were kept at 4 ^oC, the “short folding“ oligonucleotides were prepared immediately prior each experiment. After incubation with the indicated concentrations of Cdc13-DBD, the DNA-protein complexes were resolved using 6% polyacrylamide gel as described in Materials and Methods. The graph visualizes quantification of shifted probe, mean ± s.d., n = 2 (ONG1, ONG9) and n = 3 (ONG11) independent replicas. *$p<0.05$.

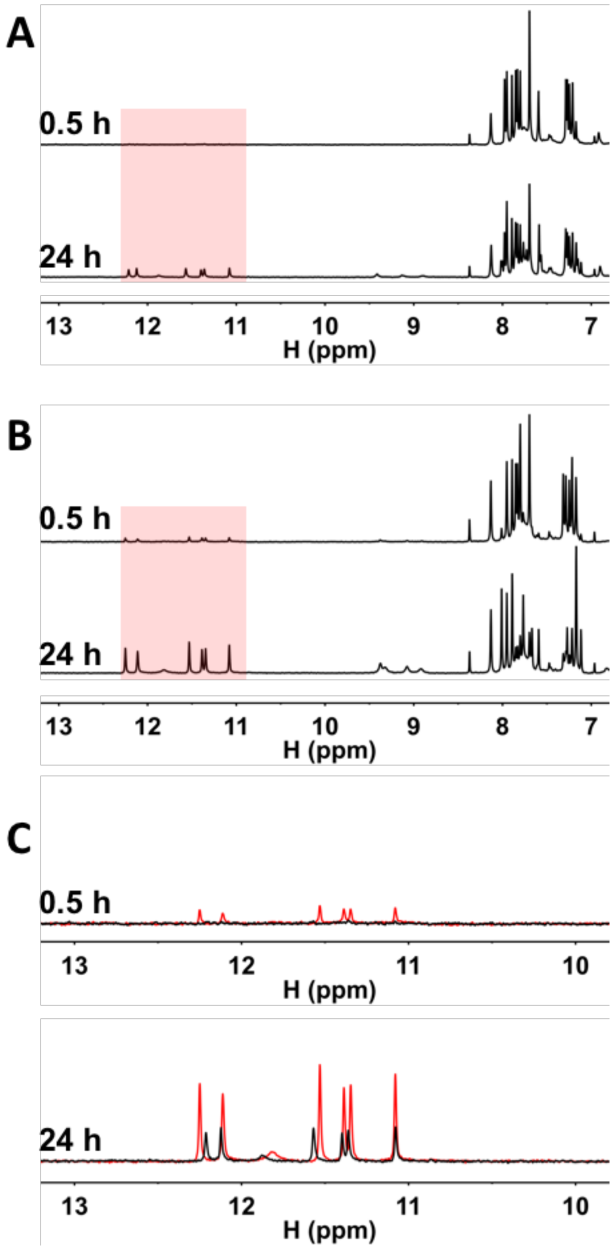

Supplementary Figure S8. **5'-terminal phosphate moiety slows down G-hairpin folding**. Imino region of ^1H 1D NMR spectra of ONG11 with 5'-terminal $-PO_3H$ (A) and 5'-terminal -OH (B) acquired as a function of time (indicated). Imino regions of the spectra reporting on a population of the folded species (G-hairpin) are highlighted (red box). (C) Overlay of imino region of the ^1H 1D NMR spectra of ONG11 with 5'-terminal $-PO_3H$ (black) and 5'-terminal -OH (red). The spectra were acquired using p3919 pulse sequence (standard Bruker library) in K^+S buffer at 25 °C.

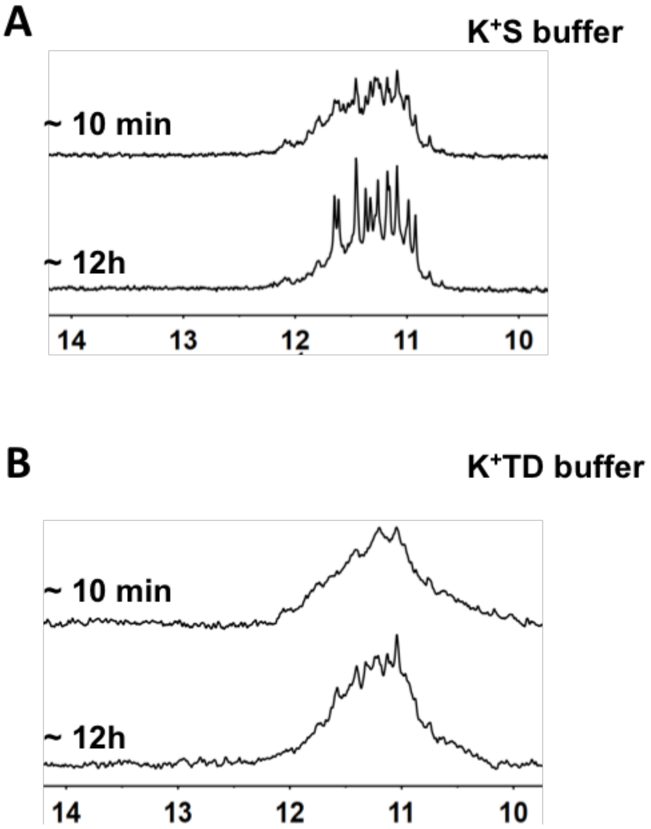

Supplementary Figure S9. Impact of buffer composition on appearance of NMR spectra. Imino regions of ^{1}H 1D NMR spectra of ONG11 acquired in $K^{+}S$ (A) and $K^{+}TD$ (B) buffer. The spectra in A) and B) were acquired using zggpw5 and 1-1 echo pulse sequence, respectively, at 25ºC.

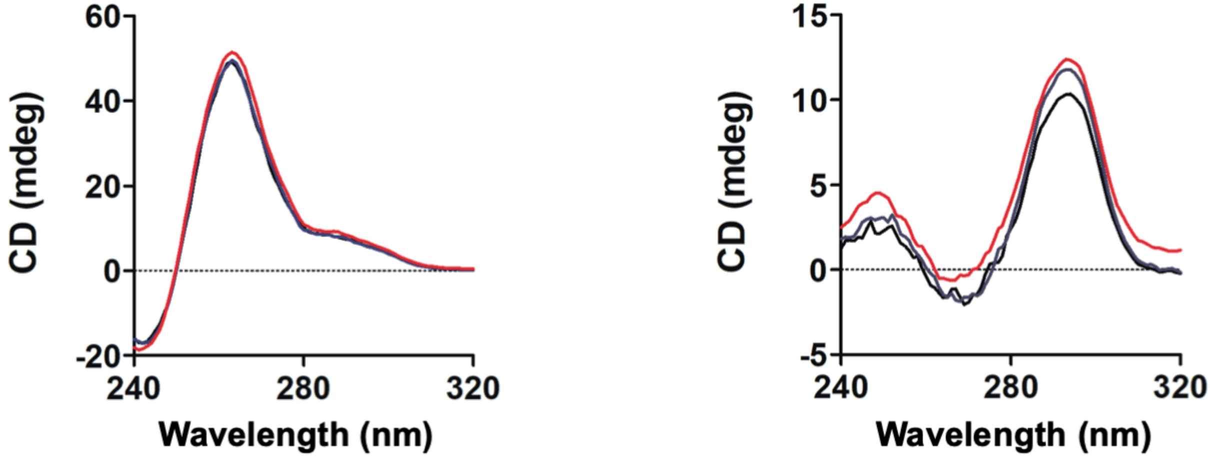

Supplementary Figure S10. Cdc13 does not induce denaturation of G4 structure. **Left:** CD spectra of ONG1. **Right**: CD spectra of ONG9. Control samples with DNA alone is indicated in red. Samples with Cdc13-DBD:ONG in 1:1 ratio were recorded immediately after Cdc13-DBD addition (blue) and after 30 min (black).
